# Plant-Derived Exosomes in Aesthetic Medicine

**DOI:** 10.34133/bmr.0397

**Published:** 2026-07-23

**Authors:** Ranchen Chen, Sidun Wei, Xin Dan, Songjie Li, Han Chen, Pu Yang, Xiangjun Liu, Tong Li, Lanjie Lei, Yang Li, Xing Fan

**Affiliations:** ^1^ Department of Plastic and Reconstructive Surgery, Xijing Hospital, Fourth Military Medical University, Xi’an 710032, China.; ^2^ Department of Burns and Plastic Surgery, The 909th Hospital, School of Medicine, Xiamen University, Zhangzhou 363000, China.; ^3^ Key Laboratory of Artificial Organs and Computational Medicine in Zhejiang Province, Institute of Translational Medicine, Zhejiang Shuren University, Hangzhou 310015, China.

## Abstract

The global cosmetic surgery industry is actively seeking alternatives that can overcome the limitations of traditional animal-derived exosomes, which face clinical translation bottlenecks including high costs, immunological risks, and zoonotic disease hazards. Plant-derived exosomes (PLDEs) offer a promising attractive “green” nanotechnology platform owing to their inherent safety, minimal immunogenicity, and suitability for scalable manufacturing. Their core advantage lies in delivering plant-specific bioactive compounds (such as small RNAs and secondary metabolites) that target multiple key signaling pathways, including skin aging, pigmentation, inflammation, and regeneration, through cross-species regulatory mechanisms. This review critically synthesizes PLDE biological basis, isolation methods, and evidence for antiaging, skin brightening, and regenerative efficacy. It delineates how surface functionalization, targeted drug delivery, and biomaterial integration (hydrogels and microneedles) optimize PLDE targeting. Finally, it addresses current challenges in clinical translation and standardization, offering a forward-looking perspective on the development of next-generation PLDE-based therapies for minimally invasive aesthetic applications.

## Introduction

Since the early 21st century, the global cosmetic surgery market has grown rapidly, driven primarily by nonsurgical minimally invasive and noninvasive procedures. Projected to expand at an average annual rate of approximately 7%, the market reflects rising consumer demand for safe, efficient, and quick-recovery aesthetic solutions. This expansion has not only spurred the exploration of innovative therapies but also shifted focus toward active delivery systems that combine high biocompatibility and natural origins.

Against this backdrop, extracellular vesicles (EVs) hold substantial promise for applications in regenerative medicine and dermatology as key carriers of intercellular communication [1]. Acting as versatile carriers, exosomes transport functional cargo, including proteins, nucleic acids, and lipids, to regulate essential physiological processes such as immune modulation and tissue repair. Exosomes derived from human mesenchymal stem cells (MSC-exosomes) have shown considerable promise in skin regeneration. Their demonstrated efficacy in enhancing collagen synthesis, stimulating fibroblast proliferation, and accelerating wound healing has positioned them as a leading therapeutic candidate in this field [2]. Nevertheless, the clinical application of animal-derived exosomes is constrained by several limitations originating from their biological source. Key hurdles include important manufacturing expenses, concerns regarding immunogenicity, and the risk of transmitting pathogens.

To overcome these limitations, plant-derived exosomes (PLDEs) have emerged as a sustainable “green” nanotechnology platform [3]. Apart from sharing the lipid bilayer structure and transport capacity of mammalian exosomes, PLDEs offer several advantages, including low immunogenicity, excellent biocompatibility, feasible large-scale production, and abundant plant-specific antioxidant and anti-inflammatory compounds [4]. Compared with synthetic nanocarriers (e.g., liposomes), PLDEs exhibit lower cytotoxicity and higher biodegradability, and their naturally occurring surface functional molecules may confer unique targeting and penetration capabilities. The antioxidant, anti-inflammatory, and melanogenesis-inhibiting activities of PLDEs are attributed to their regulatory effects on well-defined pathways such as nuclear factor erythroid 2-related factor 2 (Nrf2), nuclear factor κB (NF-κB)/mitogen-activated protein kinase (MAPK), and microphthalmia-associated transcription factor (MITF)/tyrosinase, providing a mechanistic foundation for their diverse applications in skin rejuvenation, whitening, and repair [5]. Furthermore, their robust carrier properties enable the efficient encapsulation and delivery of active ingredients, thereby markedly improving drug stability and bioavailability [6].

**Fig. 1.**
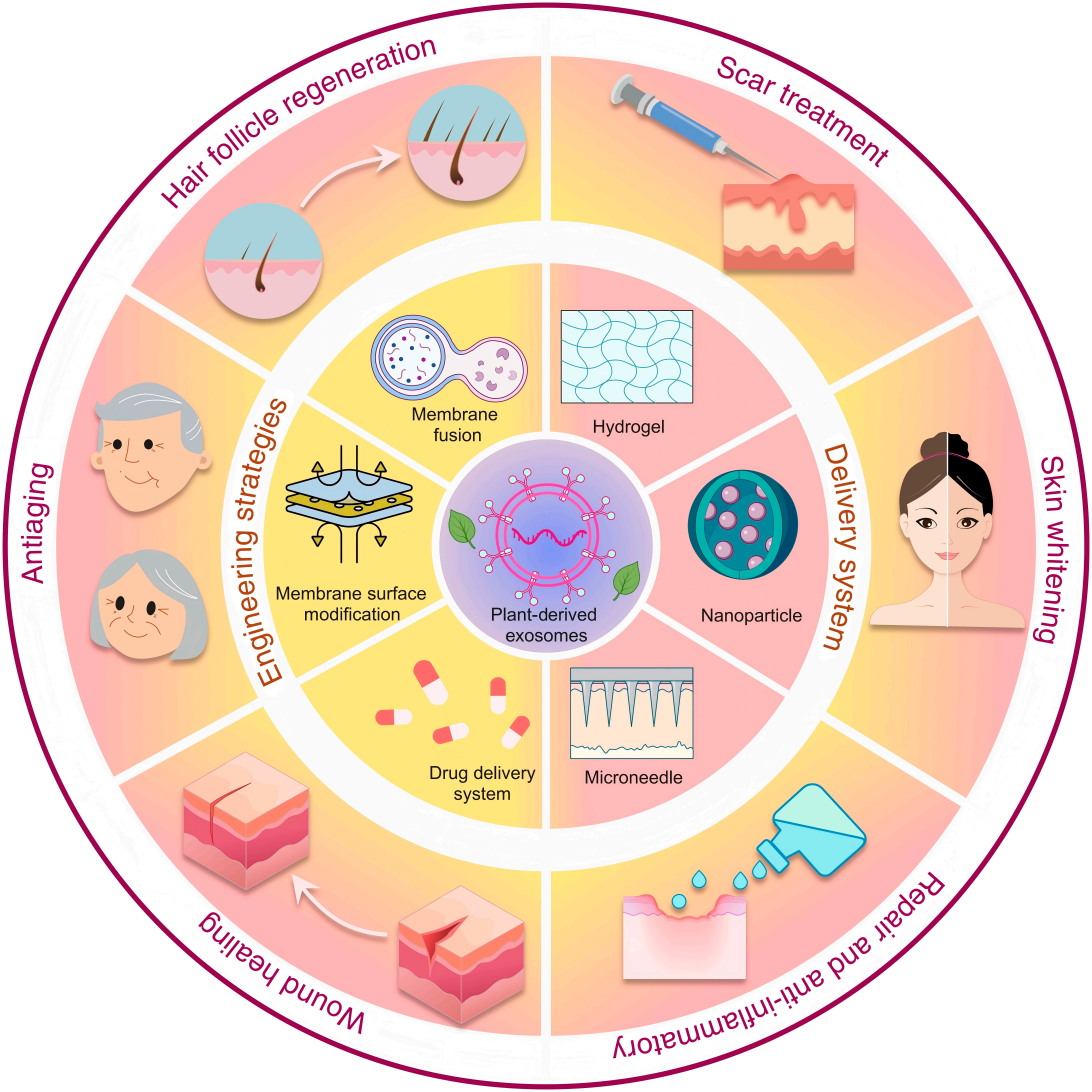
Plant-derived exosomes provide a safe, scalable nanotechnology platform for aesthetic medicine. They effectively target skin aging, pigmentation, and hair regeneration pathways through bioactive compound delivery. Surface engineering and biomaterial integration optimize their therapeutic efficacy for minimally invasive cosmetic applications.

Despite recent progress, PLDE research remains in its infancy and faces multiple hurdles, including nonstandardized isolation methods, ambiguous metabolic pathways, insufficient long-term safety evaluations, and limited clinical data. To address these gaps, this review systematically elucidates the biological properties, isolation procedures, and engineering strategies of PLDEs. We critically evaluate their mechanisms of action and research milestones in antiaging, skin whitening, barrier repair, wound healing, and hair follicle regeneration. We further explore advanced delivery platforms combining PLDEs with novel biomaterials. By analyzing existing evidence and identifying core bottlenecks, this study delineates feasible pathways to guide the informed advancement of PLDEs toward their clinical application in cosmetic surgery (Fig. 1).

## Biogenesis, Composition, and Uptake of Plant Exosomes

### Biogenesis of PLDEs

PLDEs are characterized by a lipid bilayer structure and a nanoscale size (approximately 30 to 150 nm), consistent with their classification as EVs released by plant cells. PLDEs are predominantly secreted via the endosomal system and multivesicular body (MVB) pathway. These vesicles harbor a diverse cargo of plant-specific bioactive molecules, encompassing small RNAs (e.g., microRNAs [miRNAs]), metabolites (e.g., flavonoids and terpenoids), and bioactive lipids. Current research suggests that the biogenesis of PLDEs primarily occurs through 3 pathways: vacuolar, MVB, and exocytic positive organelle (EXPO) pathways [7,8]. The MVB pathway, a key route for animal exosome generation, is critical for the biogenesis of PLDEs, serving as a fundamental biosynthetic route in plant cells [9–12]. The process commences with plasma membrane invagination to generate early endosomes. These early compartments then mature, engage with the trans-Golgi network, and evolve into MVBs. MVBs encapsulate numerous cargo-laden intraluminal vesicles, which contain active components such as RNA, DNA, and lipids. The fusion of MVBs with the plasma membrane results in the exocytosis of these laden intraluminal vesicles, which are subsequently secreted as PLDEs into the extracellular space [13,14]. The vacuolar pathway constitutes a key defense mechanism in plants, specifically activated during responses to fungal pathogen challenge. Upon infection, triggered vacuole–plasma membrane fusion mediates the secretion of preformed defensive compounds, including hydrolytic enzymes and specific proteins, mounting a direct countermeasure against the invading pathogen [15]. Research has identified PLDEs within the central vacuoles of grapefruit epidermal cells formed by fusion of mature MVB complexes during early developmental stages, suggesting a potential link between the vacuolar and MVB pathways [16]. Cui et al. [17] established that the central vacuole derives from the transformation of MVBs into small vesicles and their ensuing fusion, thereby elucidating a fundamental ontogenetic link between the MVB and vacuolar pathways. In plants, EXPO serves as an unconventional double-membrane organelle that mediates direct exocytosis to the cell wall via plasma membrane fusion, enabling the secretion of single-membrane vesicles including exosomes [18]. The relative contribution of distinct biosynthetic pathways to PLDE formation varies with the plant source and its physiological context, which explains, at least in part, the observed functional heterogeneity among PLDE populations.

### Composition of PLDEs

#### Proteins

The protein composition of PLDEs is crucial for defining their structure and function. Pinedo et al. [19] identified penetration 1 (PEN1), PEN3, and tetraspanin-8 (TET-8) as specific markers for plant exosomes. The homology of TET-8 to mammalian exosome markers (CD63, CD81, and CD9), coupled with its colocalization with intracellular vesicular compartments, positions it as a defining surface marker for PLDEs [20]. Compared with mammalian exosomes, PLDEs generally exhibit lower protein concentrations, with their proteomes dominated by cytoplasmic and transmembrane proteins [21]. Zhang et al. [22] analyzed ginger-derived PLDEs and identified multiple membrane proteins, including actin, proteases, aquaporins, and chloride channels. These proteins may participate in regulating the endocytosis and biological functions of PLDEs. Notably, PLDE surface proteins mediate cellular uptake through specific molecular interactions. The internalization of garlic-derived exosomes into HepG2 cells is facilitated via the binding of their surface lectin-like proteins to the CD98 glycoprotein, a mechanism substantiated by the significant (~47%) uptake inhibition observed following receptor blockade [23].

#### Lipids

Lipids not only form the backbone of the PLDE lipid bilayer but also significantly influence their stability, function, and cellular uptake capacity. The lipidome of PLDEs is characterized by a rich complement of both structural and signaling lipids. Key constituents range from glycerophospholipids and neutral lipids to abundant plant-specific galactolipids [24]. Although the lipid composition of PLDEs exhibits significant heterogeneity depending on the botanical source, phosphatidic acid consistently emerges as the predominant lipid species. Phosphatidic acid facilitates diverse biological processes, including cell proliferation, signal transduction, and the cellular internalization of PLDEs. By contrast, phosphatidylethanolamine is primarily instrumental in modulating membrane curvature [25]. In contrast to mammalian exosomes, PLDEs lack cholesterol, which may influence their membrane fusion properties within mammalian cells [26]. The distinct lipid profile of PLDEs directly underlies their tissue-specific biodistribution. For instance, ginger-derived exosome-like nanovesicles exhibit a pronounced tropism for and are selectively internalized by gut-resident *Lactobacillus* species. This selective uptake is closely associated with their unique lipid composition [27]. These findings point to the possibility of directing PLDEs to particular tissues or cells as a means to modulate their lipid profile.

#### Nucleic acids

Nucleic acids, biological macromolecules comprising DNA or RNA, carry genetic information and enable gene expression or gene regulation within cells. They are inherently used in applications such as gene therapy, RNA interference, and vaccine development [28]. As key mediators of intercellular communication, PLDEs transport a diverse nucleic acid cargo comprising DNA and multiple RNA species. Among these cargoes, miRNAs, small noncoding RNAs of ~22 nucleotides, posttranscriptionally regulate gene expression through mRNA translational repression or degradation [29,30]. Zhang et al. [31] attributed the bioactivity of most therapeutic PLDEs to the function of their encapsulated miRNAs. Notably, PLDEs significantly differ in nucleic acid composition from their source tissues. For instance, compared with ginger tissue, ginger-derived PLDEs contain a higher abundance of miRNA transporters while exhibiting a significantly lower content of tRNAs [32]. The published literature has confirmed that the nucleic acid components carried by PLDEs influence recipient cell functions through cross-species regulatory mechanisms. For example, miRNAs encapsulated within grape-derived exosomes exert anti-inflammatory effects by delivering their cargo to human digestive tract cells, where they down-regulate the expression of key proinflammatory mediators such as NF-κB, interleukin-6 (IL-6), IL-8, and tumor necrosis factor-α (TNF-α), consequently attenuating lipopolysaccharide (LPS)-induced inflammatory responses [33]. Similarly, osa-miR164d in ginger-based exosome-like nanoparticles (ELNs) exerts immunomodulatory effects by suppressing NF-κB signaling and regulating macrophage polarization, which led to the development of bionic exosomes for the targeted delivery of this miRNA for treating inflammatory skin diseases [28]. Furthermore, PLDEs show promise in antiviral therapy, wherein miRNA-mediated suppression of key viral proteins (nonstructural protein 12 and spike genes) effectively alleviates inflammation and cellular damage, thus revealing insights and novel pathways for the treatment of COVID-19 and virus-associated skin diseases [32].

#### Secondary metabolites

Secondary metabolites are small-molecule compounds synthesized by plants during growth, development, or in response to environmental stress. These molecules, primarily phenolics, terpenoids, alkaloids, and sulfur-containing compounds, play crucial roles in defense against pests and diseases, stress resistance, signal transduction, and plant morphogenesis [34]. Recent studies have indicated that secondary metabolites in PLDEs predominantly accumulate within membrane structures as lipophilic compounds, such as terpenoids, flavonoids, and phenolic acids. By contrast, relatively hydrophilic secondary metabolites are less readily actively packaged into PLDEs [35,36]. Bioactive secondary metabolites have been identified in various PLDEs. For example, ginger-derived PLDEs contain active components such as 6-gingerol and 6-zingerone [22]. The bioactivity of lemon PLDEs is attributed to constituents such as hesperidin, naringin, neohesperidin, and limonin, which confer antioxidant and anti-inflammatory properties. Among these, hesperidin exerts a specific antimelanogenic effect by down-regulating tyrosinase, pointing to its application in managing skin pigmentation [37]. Secondary metabolites in PLDEs not only possess inherent biological activity but also enhance cellular uptake. For example, Xu et al. [38] found that β-glucan in oat-derived PLDEs promotes dose-dependent uptake of PLDEs by microglia. Notably, certain PLDEs exert biological effects by regulating key signaling pathways despite a lack of expected active components. Although devoid of standard saponins, Chinese yam PLDEs retain the capacity to stimulate osteoblast differentiation, specifically through activation of the bone morphogenetic protein-2 (BMP-2)/p38/Runt-related transcription factor 2 (Runx2) pathway [39]. BMP-2 orchestrates key processes in skin repair and hair follicle biology. In hair follicle stem cells, it activates a transcriptional program involving phosphatase and tensin homolog up-regulation, which consequently stimulates autophagy and initiates differentiation, culminating in enhanced tissue regeneration [40]. These findings suggest that PLDEs exert multifaceted effects through complex signaling networks.

### Uptake mechanism of PLDEs

PLDEs exhibit a broad cellular tropism encompassing malignant cells (breast, lung, colon, and glioma), lymphocytes (T and B cells), various neural cell types (neurons, microglia, astrocytes, and microvascular endothelial cells), and normal human skin keratinocytes (e.g., HaCat cells) [41]. The cellular uptake of PLDEs involves multiple routes, using 3 principal mechanisms: (a) membrane fusion: This mechanism involves the direct merger of the PLDE bilayer with the cellular plasma membrane, thereby discharging its internal payload into the cytoplasm, unassisted by classical endocytic machinery. A plausible mechanism for this process involves membrane docking and fusion mediated by the SNARE protein family, with vesicle transport regulated by Rab guanosine triphosphatases, resulting in a semifused stalk intermediate structure [42,43]; (b) endocytic pathways: PLDEs can be internalized through energy-dependent, temperature-sensitive endocytosis. The process entails the engagement of multiple molecular pathways, including clathrin-mediated endocytosis, lipid-raft-dependent internalization, and caveolar uptake [42–45]; (c) Receptor-mediated signal activation: Lectin-like proteins bind to corresponding receptors on target cell membranes (e.g., CD98), thereby activating downstream signaling pathways to regulate cellular functions [23,28,42]. These mechanisms not only promote the internalization of PLDEs but also confer targeted properties upon them, providing a molecular foundation for subsequent engineering modifications such as ligand display.

## Isolation of PLDEs

Robust methodologies for isolating and purifying PLDEs are essential to support their detailed mechanistic investigation and potential clinical translation. The choice of separation method, which exploits differences in key physicochemical properties of PLDEs (e.g., size, density, solubility, and surface antigenicity), critically determines the yield, purity, and retained biological activity of the isolated vesicles. This section systematically reviews primary separation strategies and summarizes their advantages, limitations, and translational potential.

### Differential ultracentrifugation

Differential ultracentrifugation (dUC) is a predominant method for isolating PLDEs. This technique uses a stepwise increase in centrifugal force to sequentially pellet particles based on differential sedimentation rates [46]. The process begins by removing cellular debris and large vesicles at lower speeds, ultimately isolating purified PLDEs via a final high-speed ultracentrifugation step [25]. To enhance purity, the precipitate is often resuspended and subjected to density gradient ultracentrifugation using a sucrose pad. This purification leverages the density differences between PLDEs and contaminants such as soluble proteins [26]. dUC has been successfully applied to isolate PLDEs from plants such as kudzu root, apple, Chinese yam, and *Ganoderma lucidum* owing to its relatively simple operation and lack of complex reagents [39,47,48]. However, this method has significant limitations. The immense shear forces generated via ultracentrifugation may cause physical damage to the vesicle membrane structure, compromising its integrity and function. In addition, coaggregation often results in nonvesicular protein aggregates in the final product, reducing its purity. Furthermore, the process is time consuming and requires expensive equipment, making it unsuitable for large-scale production [49].

### Density gradient centrifugation

Density gradient centrifugation is a crucial optimization strategy for dUC. In this process, samples are placed atop a preformed density gradient medium (e.g., sucrose or ioxaglate), and components within the sample are migrated to gradient zones matching their own density via ultracentrifugation, thereby achieving high-purity separation [50]. This technology separates PLDEs from contaminants with significantly different densities, such as protein polymers and organelle fragments. It has been applied to extract well-preserved PLDEs from plants such as bitter melon, edible fungi, ginseng, and *Artemisia annua* [43,44,51]. The purity of PLDEs obtained via density gradient centrifugation is significantly superior to those obtained using conventional dUC. However, the process is cumbersome, requires high-precision gradient preparation, and yields limited output, which restrict its application to small-scale production.

### Polyethylene glycol precipitation

Polyethylene glycol (PEG) precipitation, a separation method based on changes in solubility, involves the hydrophilic polymer PEG competing for water molecules in the solution, disrupting the solvation layer of PLDEs. This phenomenon reduces their solubility, inducing vesicle aggregation and precipitation. This method typically involves pretreating plant extracts via low-speed centrifugation, followed by mixing with a specific concentration of PEG solution and incubating overnight at low temperatures, with precipitated PLDEs collected through low-speed centrifugation. It has been used to isolate ELNs from blueberries and *Physalis* [52,53]. The main advantages of PEG precipitation are its low cost, simple operation, and lack of requirement for specialized ultracentrifugation equipment, rendering it highly suitable for preliminary research in standard laboratories. However, a drawback is the nonspecific precipitation of impurities (such as host plant proteins and nucleic acids), which compromises purity. Furthermore, PEG molecules can be adsorbed onto the surface of PLDEs, interfering with downstream functional studies or cellular uptake experiments [54].

### Size-based isolation

The core principle underlying such methods is the nanoscale dimension of PLDEs, primarily encompassing ultrafiltration and size exclusion chromatography (SEC) [55]. Ultrafiltration is a tangential flow filtration technique that uses semipermeable membranes with defined molecular weight cutoffs or pore sizes (typically 100 to 300 kDa or 10 to 300 nm) by retaining them while allowing smaller molecules to pass through via centrifugation or pressurization. For example, when isolating *Pueraria* root PLDEs, Wu et al. [56] used an initial clarification through a 0.22-μm filter to deplete large particles, prior to sample concentration via ultrafiltration. This method is rapid, gentle, easily scalable, and preserves vesicle activity. However, the membrane pores can become clogged by viscous substances or particles in the sample, resulting in reduced efficiency and vesicle loss. SEC is a liquid chromatography technique based on hydrodynamic volume. When samples are passed through a porous gel packing material, large PLDEs cannot enter the gel pores and thus flow rapidly through the interstitial spaces, eluting first. By contrast, small molecular contaminants can enter the pores, travel a longer path, and elute more slowly. You et al. [57] used SEC to isolate high-purity PLDEs from cabbage. The primary advantage of SEC is its mild separation conditions, which effectively preserve the natural biological activity and structural integrity of PLDEs. Furthermore, the elution buffer is physiologically compatible, facilitating downstream applications. Its disadvantages include a limited sample throughput and stringent requirements for preclarification of the initial sample.

### Immunoaffinity capture

Immunoaffinity capture is a high-purity isolation technique based on antigen–antibody specific reactions [58]. It utilizes surface-specific or enriched membrane proteins (such as members of the transmembrane protein family) as targets, achieving specific capture through immobilized antibodies (often conjugated to magnetic beads or chromatographic column matrices) [59]. For example, He et al. [20] successfully purified TET-8-positive PLDEs from various plant sap extracts using antibody-coated magnetic beads targeting the extracellular domain (EC2) of the plant-specific transmembrane protein TET-8 [20]. This method offers superior purity over other physical approaches, making it suitable for molecular mechanism studies and diagnostic biomarker discovery. However, its application faces several bottlenecks. First, the number of validated specific surface markers for PLDEs remains extremely limited. Second, antibodies are costly, and harsh elution conditions (e.g., low pH) may compromise vesicle integrity and biological activity. Finally, this method can only isolate PLDE subpopulations expressing specific antigens, failing to reflect the overall vesicle composition.

Future trends in PLDE separation technology will prioritize standardized workflows, combining the advantages of multiple methods (such as SEC coupled with ultrafiltration) to balance purity and yield, and explore novel methods to reduce the cost of highly specific capture, for example, Xu et al. [60] proposed an innovative method called multiexosomics, which enables simultaneous analysis of extracellular metabolites and proteins. This method utilizes aptamer-functionalized magnetic beads to capture exosomes targeting the CD63 protein and uses double-strand specific deoxyribonuclease to separate and purify the exosomes, demonstrating high sensitivity, specificity, and anti-interference capabilities. The resolution of these technical challenges is pivotal for enabling the successful clinical translation of PLDEs, particularly in cosmetic surgery.

## Strategies for PLDE Engineering

Similar to animal-derived or synthetic nanocarriers, PLDEs deliver bioactive substances to target cells and regulate intercellular communication, thereby demonstrating potential as therapeutic platforms. However, inherent drawbacks, such as suboptimal intrinsic targeting and constrained therapeutic potency, have spurred the development of diverse engineering strategies aimed at augmenting their functionality. For instance, PLDEs exhibit superior biocompatibility and lower immunogenicity over synthetic nanocarriers (e.g., liposomes and polymeric nanoparticles) but face challenges in achieving precise surface functionalization and consistent drug loading because of the lack of genetically modifiable parental cells. Thus, strategies such as surface engineering, cargo loading, and membrane fusion are used [61]. The choice of engineering strategy often depends on specific application objectives. While surface modification and membrane fusion are used to enhance stability and targeting capabilities, drug loading focuses on improving the encapsulation efficiency and delivery efficacy of active ingredients. Although PLDE research remains in its infancy, we can draw on experience from animal exosome and artificial nanoparticle research. The synergistic application of multiple techniques has demonstrated considerable potential in the development of multifunctional PLDE delivery systems [62–64].

### Membrane surface modification

**Fig. 2.**
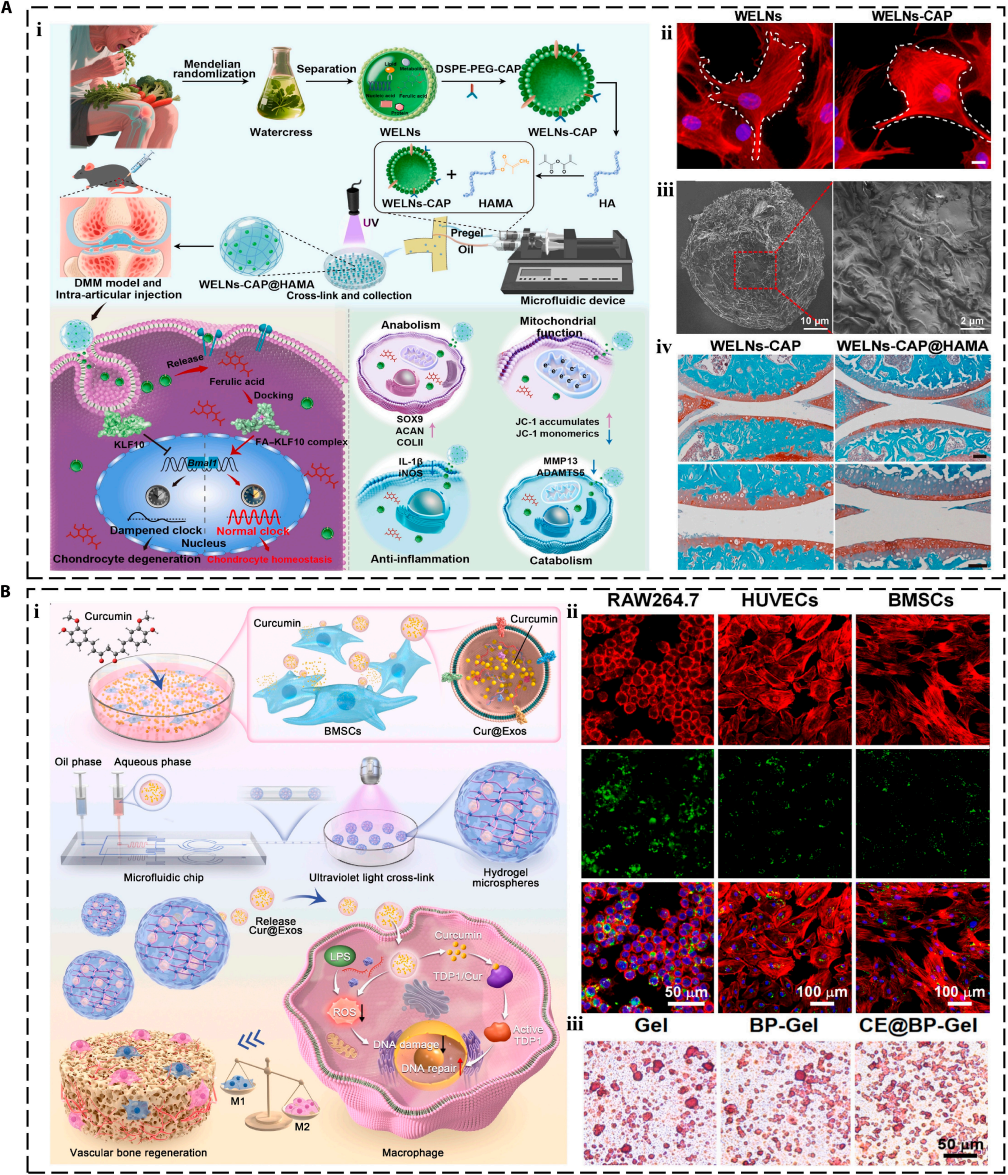
Preparation and mechanism of engineered plant-derived exosomes (PLDEs). (A) (i) Schematic of the engineered watercress-derived exosomes-like nanovesicles modified with chondrocyte affinity peptide (WELNs-CAP) and WELNs-CAP encapsulated in methacrylic anhydride modified hyaluronic acid (WELNs-CAP@HAMA). (ii) Confocal imaging of cytoskeletal reorganization in cells treated with WELNs versus WELNs-CAP. Scale bar, 10 μm. (iii) Scanning electron micrographs of freeze-dried WELNs-CAP@HAMA microgels. (iv) SF staining results of WELNs-CAP and WELNs-CAP@HAMA. Scale bar, 100 μm. Adapted and reprinted with permission from Ref. [68]. Copyright 2025 The Authors. Publishing services by Elsevier B.V. on behalf of KeAi Communications Co. Ltd. (B) (i) Schematic of curcumin-loaded exosomes (Cur@Exos) embedded in gelatin methacryloyl (GelMA) hydrogel microspheres. (ii) Fluorescence micrographs of exosome internalization in RAW264.7 macrophages, human umbilical vein endothelial cells (HUVECs), and bone marrow mesenchymal stem cells (BMSCs) following coculture with curcumin-loaded mesenchymal-stem-cell-derived exosomes encapsulated in bisphosphonate-modified GelMA hydrogel (CE@BP-Gel) microspheres. (iii) Tartrate-resistant acid phosphatase staining. Adapted and reprinted with permission from Ref. [80]. Copyright 2025 The Authors. Published by Elsevier Ltd.

The most widely utilized strategy for modifying nanocapsule membranes is biological functionalization, with its core objective being to enhance pharmacokinetic properties and achieve tissue-specific targeting [65–67]. Membrane surface modification serves as a primary means to accomplish pharmacokinetic optimization and site-directed targeting in PLDE therapeutics (Fig. 2A) [68]. Given the difficulty of genetically engineering plant parental cells, research has primarily focused on directly modifying isolated PLDEs [69,70]. The primary direct modification strategies include (a) covalent linkage—utilizing cross-linkers such as 1-ethyl-3-(3-dimethylaminopropyl)carbodiimide (EDC)/*N*-hydroxysuccinimide (NHS), targeted ligands can be covalently anchored to the membrane surface of PLDEs. The underlying molecular mechanism involves the activation of carboxyl groups on the PLDE membrane or ligands, forming stable amide bonds. For example, heparin bridging of cyclic RGD peptides targeting α_v_β_3_ integrin to citrus PLDEs significantly enhances their tumor-targeting capability [71]. Adapters (such as hemagglutinin subunit 1 targeting human epidermal growth factor receptor 2) can also confer cell-specific recognition capabilities to PLDEs through covalent linkage [72]. (b) Hydrophobic insertion and click chemistry—inserting hydrophobic-modified molecules (e.g., 1,2-distearoyl-*sn*-glycero-3-phosphoethanolamine [DSPE]–PEG–ligand) into the lipid bilayer, followed by secondary coupling via click chemistry, enables complex surface engineering. For example, presenting the targeted aptamer R11-3 to grapefruit ELNs via this strategy enhances its ability to cross the blood–brain barrier, and folate (FA)-modified ginger ELNs specifically target FA-receptor-positive tumor cells by leveraging this well-characterized ligand–receptor recognition pathway [73–75]. (c) PEGylation-surface modification with PEG molecules shields PLDEs, reducing nonspecific uptake by the reticuloendothelial system and prolonging circulation time in vivo [76]. However, PEGylation may also affect the interaction between PLDEs and cells, requiring careful consideration in practical applications. The efficacy of surface modification can be systematically validated using techniques ranging from physicochemical characterization (e.g., dynamic light scattering and zeta potential analysis) to specific ligand–receptor interaction assays (e.g., flow cytometry, surface plasmon resonance, and Western blot). Several challenges remain in this field. First, chemical modifications may compromise membrane integrity and native activity. Second, efficient and uniform modifications are difficult to achieve. Last, the absence of well-defined, specific membrane protein markers for PLDEs hinder the rational design of precise targeting strategies. Different from synthetic nanocarriers, where well-established click chemistry and PEGylation protocols exist with >80% modification efficiency, PLDE surface engineering still suffers from batch-to-batch variability and limited quantitative control.

### Cargo encapsulation

The lipid bilayer membrane and internal lumen of PLDEs offer a natural, versatile space for loading therapeutic molecules (small-molecule chemotherapeutic drugs, nucleic acids, proteins, etc.), rendering them a promising class of bio-based delivery carriers (Fig. 2B) [77–80]. Compared with synthetic nanocarriers such as liposomes and micelles, PLDEs s exhibit lower immunogenicity, higher biocompatibility, and stronger cellular uptake capacity, which contribute to their enhanced delivery efficiency and therapeutic safety [81–83]. Various drug delivery strategies based on physical, chemical, or combined physicochemical approaches have been developed to achieve efficient loading of therapeutic molecules. These strategies include physical forces (e.g., electroporation, ultrasonication, and freeze–thaw), chemical reactions (e.g., click chemistry), or combined principles to facilitate cargo internalization [82]. A comparative analysis revealed that electroporation offers high encapsulation efficiency (up to 70%) for nucleic acids but may cause vesicle aggregation and cargo degradation, ultrasonication is gentler but yields lower efficiency (30% to 50%), and freeze–thaw is simple but less reproducible. Mammadova et al. [78] successfully encapsulated curcumin into ELNs derived from tomatoes via ultrasound-assisted coincubation, significantly enhancing its anti-inflammatory activity. Itakura et al. [84] used microfluidic technology to optimize pressure parameters, enabling efficient small interfering RNA (siRNA) encapsulation in grapefruit-derived PLDEs. Beyond single-drug delivery, PLDEs can act as codelivery platforms. For instance, combining bitter-melon-derived EVs with 5-fluorouracil enhances the suppression of oral squamous cell carcinoma by inhibiting nucleotide-binding oligomerization domain, leucine-rich repeat, and pyrin-domain-containing protein 3 signaling [85]. However, achieving efficient and stable drug loading while maintaining the membrane integrity and biological activity of PLDEs remains challenging. Hydrophilic drug loading is restricted by limited bilayer permeability, resulting in low drug loading efficiency. Electroporation can cause vesicle aggregation and alter surface potential, thus compromising the natural composition and function of the drug. To address these challenges, future research should prioritize the development of innovative techniques such as microfluidic precision control and membrane-permeable peptide-assisted loading to balance drug loading efficiency with carrier integrity. Postloading, systematic evaluation of encapsulation efficiency, drug loading capacity, and in vitro release behavior via high-performance liquid chromatography and other methods is essential to lay the groundwork for subsequent functional and application studies.

### Membrane hybridization and coating

Based on biomimetic design principles, hybridizing or coating PLDEs with specific cell membranes can effectively enhance immune compatibility, prolong systemic circulation half-life, and augment tissue-targeting specificity [86]. Hybrid membrane nanovesicles integrate multiple membrane-associated functions by fusing biological membranes from different sources, enabling performance customization [87]. Compared with synthetic nanocarriers that rely on chemical modification for targeting, membrane hybridization offers lower immunogenicity and more natural targeting behaviors by leveraging native receptor–ligand interactions. For example, Zhuang et al. [88] fused bacterial outer membrane vesicles with chloroplast thylakoid vesicles to construct a hybrid system that not only targets tumors but also improves the tumor microenvironment through photocatalytic oxygen production and activates antitumor immunity, demonstrating potential as an in situ vaccine. Similarly, hybridizing ginseng-derived EV-like particles with autologous tumor cell membranes enhances antigen uptake by dendritic cells and activates specific T cells, offering a strategy for preventing tumor recurrence [89]. In addition, coating PLDEs with specific cell membranes, such as leukocyte membranes or neutrophil membranes, can confer active chemotaxis and inflammation-targeting capabilities. For example, grapefruit-derived nanocarriers coated with leukocyte membranes can target inflammatory sites by utilizing CXCR2 and lymphocyte function-associated antigen-1 receptors on the membrane [90]. Ginseng-derived ELNs coated with neutrophil membranes can deliver miRNA-182-5p specifically to the lungs, thereby alleviating sepsis-related inflammation [91]. Membrane hybridization strategies can integrate the biological functions of exogenous membranes while preserving the inherent properties of PLDEs, thereby expanding their application prospects in immunomodulation and targeted therapy. Currently, this technology still faces challenges in hybridization efficiency, product stability, and large-scale preparation. The fusion effect is typically validated using techniques such as cryo-electron microscopy and fluorescence resonance energy transfer.

### Other modification strategies

To address complex disease environments, developing intelligent PLDE delivery systems that integrate multiple engineered strategies has become a significant trend. Multifunctional high-efficiency nanocarriers can be constructed by synergistically using targeted modification, drug loading, and membrane fusion technologies. For instance, FA-modified ginger-derived nanocarriers enhance the targeted delivery and cytotoxic effects of doxorubicin (DOX) against colon cancer cells [92]. Huang et al. [93] engineered a biomimetic hybrid system through membrane fusion between grapefruit-derived exosomes and membranes isolated from gingival MSCs overexpressing CCR6. They encapsulated the immunomodulator CX5461 within this system, enabling active homing to inflammatory sites and remodeling of the immune microenvironment. Zeng et al. [94] achieved synergistic photochemotherapy and targeted treatment for breast cancer by coloading DOX and indocyanine green via π–π stacking within aloe vera vesicles, followed by conjugation of an integrin-targeting peptide. Several other engineered approaches have been reported in research. For instance, Zhuang et al. [95] pioneered the use of high-pressure homogenization technology to extract lipids (grapefruit-derived nanovesicle) from grapefruit for the fabrication of nanovectors. Their work established the capacity of grapefruit-derived nanovesicles for drug and siRNA carriage, followed by efforts to engineer surface modifications that improve targeting specificity and therapeutic delivery. In addition, the stability and controlled release properties of PLDEs can be enhanced through material composite strategies. For instance, combining olive-leaf-derived PLDEs with hyaluronic acid (HA)–tannic acid (TA) hydrogels effectively attenuates ultraviolet (UV)-induced photodamage and enhances skin regeneration [96]. Although the aforementioned synergistic strategy demonstrates promising potential, its clinical translation requires further validation. In particular, its long-term safety and large-scale preparation necessitates systematic evaluation.

## PLDE-Based Delivery Systems

### Role of PLDEs in drug delivery systems

Drug delivery systems (DDSs) aim to improve therapeutic efficacy and minimize side effects by optimizing delivery routes, controlling release rates, and targeting specific tissues [97]. While traditional synthetic nanoparticles (e.g., liposomes) are established DDS carriers, plant exosomes offer distinct advantages. Their innate lipid bilayer structure confers inherent biocompatibility and superior membrane fusion properties, positioning them as a compelling next-generation delivery platform [83]. Similar to synthetic nanoparticles, PLDEs can encapsulate hydrophilic and hydrophobic molecules while exhibiting superior biocompatibility, stability, and delivery efficiency [98]. PLDEs exhibit 2 key advantages over liposomes. One is their enhanced biocompatibility and safety. PLDEs exhibit low cytotoxicity and are less likely to induce adverse reactions such as oxidative stress. Intravenous administration of lipid-based drugs at equivalent doses can cause liver damage, whereas no similar toxicity has been observed with PLDEs [99,100]. Wang et al. [41] reported that grapefruit-derived PLDEs exhibit a superior safety profile over liposomes. Following intravenous injection at identical doses, liposomes, but not PLDEs, induce measurable liver function impairment. The other is their enhanced stability and delivery efficiency. PLDEs exhibit excellent structural rigidity, minimal immunogenicity, and efficient biological barrier crossing, contributing to extended circulation time and improved drug bioavailability [101]. For example, Liu et al. [102] found that curcumin can be loaded into grape-derived PLDEs in an amorphous form via hydrogen bonding, thereby significantly enhancing its solubility and stability.

**Fig. 3.**
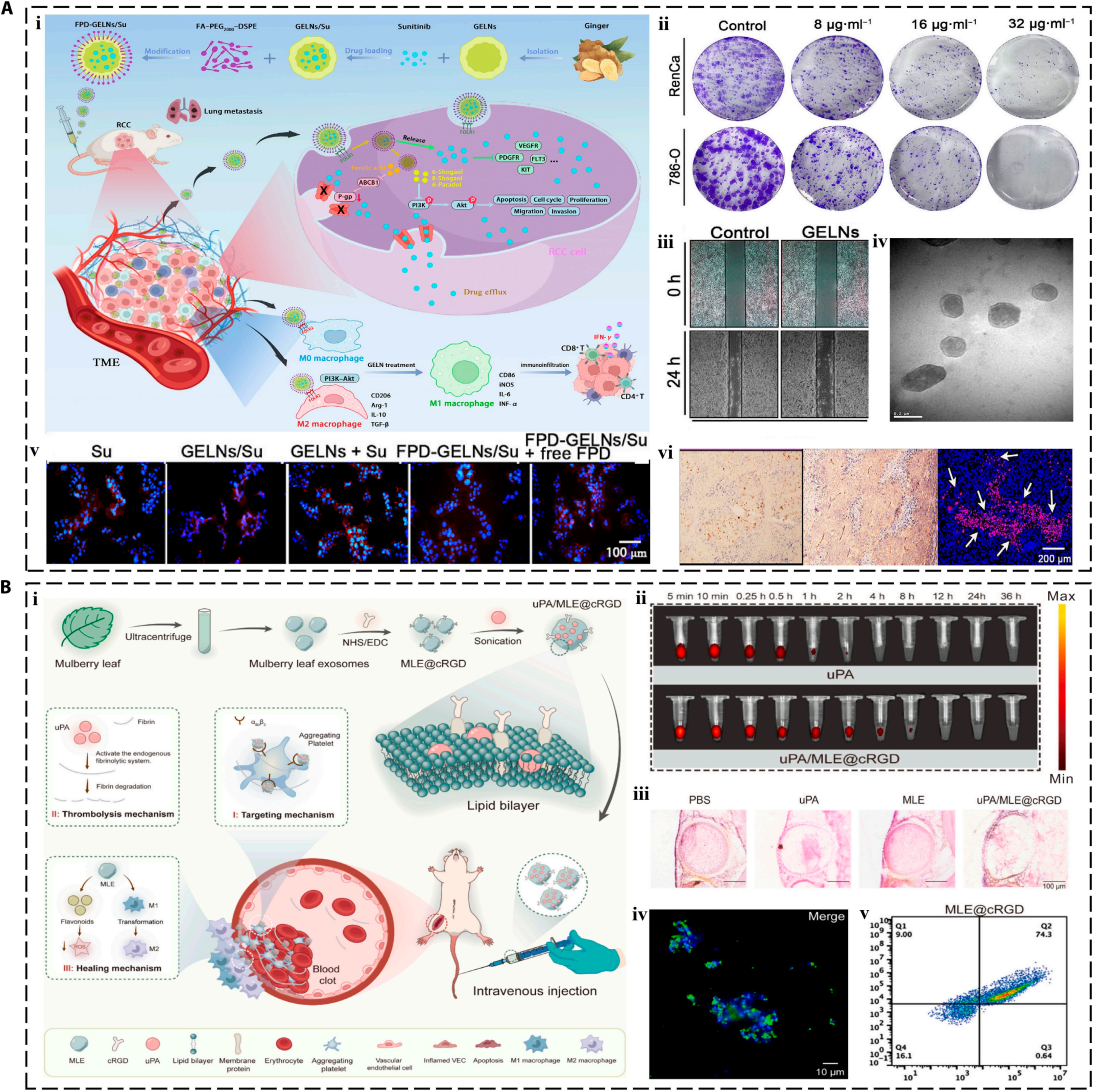
Drug delivery function of PLDEs. (A) (i) Schematic of the drug loading strategy and multifunctional therapeutic mechanisms of ginger-derived exosome-like nanoparticles (GELNs). (ii) Colony formation assay demonstrating dose-dependent suppression of proliferative capacity in cells following treatment with GELNs. (iii) Wound healing assay showing that GELNs markedly inhibit cell migration over time. (iv) Transmission electron micrograph of GELNs. (v) Fluorescence images indicate that GELNs enhance the intracellular enrichment of drugs. (vi) Immunohistochemical and terminal deoxynucleotidyl-transferase-mediated deoxyuridine triphosphate nick end labeling staining. Adapted and reprinted with permission from Ref. [103]. Copyright 2025 The Author(s). Advanced Science published by Wiley-VCH GmbH. (B) (i) Engineering strategy and multifunctional mechanism of urokinase-type plasminogen activator-loaded, cyclic arginine–glycine–aspartic acid-conjugated mulberry leaf exosomes (uPA/MLE@cRGD). (ii) In vivo blood fluorescence tracking showing prolonged circulation and sustained retention of uPA when delivered via uPA/MLE@cRGD. (iii) Hematoxylin and eosin staining of the femoral vein after 6 h of different treatments. (iv) Fluorescence colocalization imaging. (v) Flow cytometric analysis showing high cellular uptake efficiency of MLE@cRGD. Adapted and reprinted with permission from Ref. [113]. Copyright 2024 Elsevier B.V. VEGFR, vascular endothelial growth factor receptor; PDGFR, platelet-derived growth factor receptor.

PLDEs exhibit promising clinical application potential as DDS platforms (Fig. 3A) [103]. Niu et al. [104] constructed a composite DDS by conjugating DOX-loaded heparin exosomes onto the surface of grapefruit-derived PLDEs, thereby enhancing drug loading capacity and improving efficacy against glioblastoma. In tumor combination therapy, Zeng et al. [94] utilized aloe-derived vesicles to coload indocyanine green and DOX, achieving synergistic photochemotherapy effects against breast cancer. Hybrid nanoparticles for cosmetic surgery were constructed by encapsulating CX5461 within grapefruit-derived PLDEs and then coating them with CCR6-enriched membranes derived from gingival MSCs; these nanoparticles can be intravenously injected to specifically target inflammatory regions in autoimmune skin diseases, thereby restoring the imbalanced immune microenvironment [93].

PLDEs utilize diverse delivery routes: Intravenous injection remains the most common method owing to its enhanced bioavailability, rapid pharmacological onset, and circumvention of hepatic first-pass metabolism [105]. Conversely, oral administration is a noninvasive route of drug delivery, offering a lower risk of infection, enhanced gastrointestinal permeability, and hepatic clearance avoidance [105,106]. Oral administration of PLDEs enables the rapid onset of drug action [75,107,108]. Transdermal drug delivery involves PLDE penetration of the skin barrier through 2 pathways: the follicular and stratum corneum pathways. Mechanistically, when PLDEs are applied topically to the skin, they permeate through the “lipid-rich channel” coating on the follicles [109]. PLDEs structurally resemble liposomes, and their transdermal delivery capability via this route has been demonstrated [110]. PLDEs can exploit both transcellular and intercellular routes for stratum corneum permeation. This capability is attributed to their biomimetic lipid composition, which enables direct fusion with keratinocyte membranes. Highly lipophilic broccoli-derived PLDEs efficiently fuse with and deliver fluorescent cargo into keratinocytes, underscoring their promise as transdermal carriers [111]. This property has been utilized for transdermal gene therapy in cutaneous melanoma [112]. In summary, PLDEs, as a multifunctional and highly efficient DDS, hold broad application prospects in cosmetic surgery (Fig. 3B) [113].

### Advanced delivery systems of PLDEs

Although PLDEs demonstrate significant potential in tissue repair and regeneration, their rapid clearance in vivo limits sustained retention and release at target sites [114–116]. Combining PLDEs with biomaterials to construct composite delivery systems can effectively overcome this limitation, enabling controlled release and enhanced efficacy of PLDEs [117].

#### Hydrogels

**Fig. 4.**
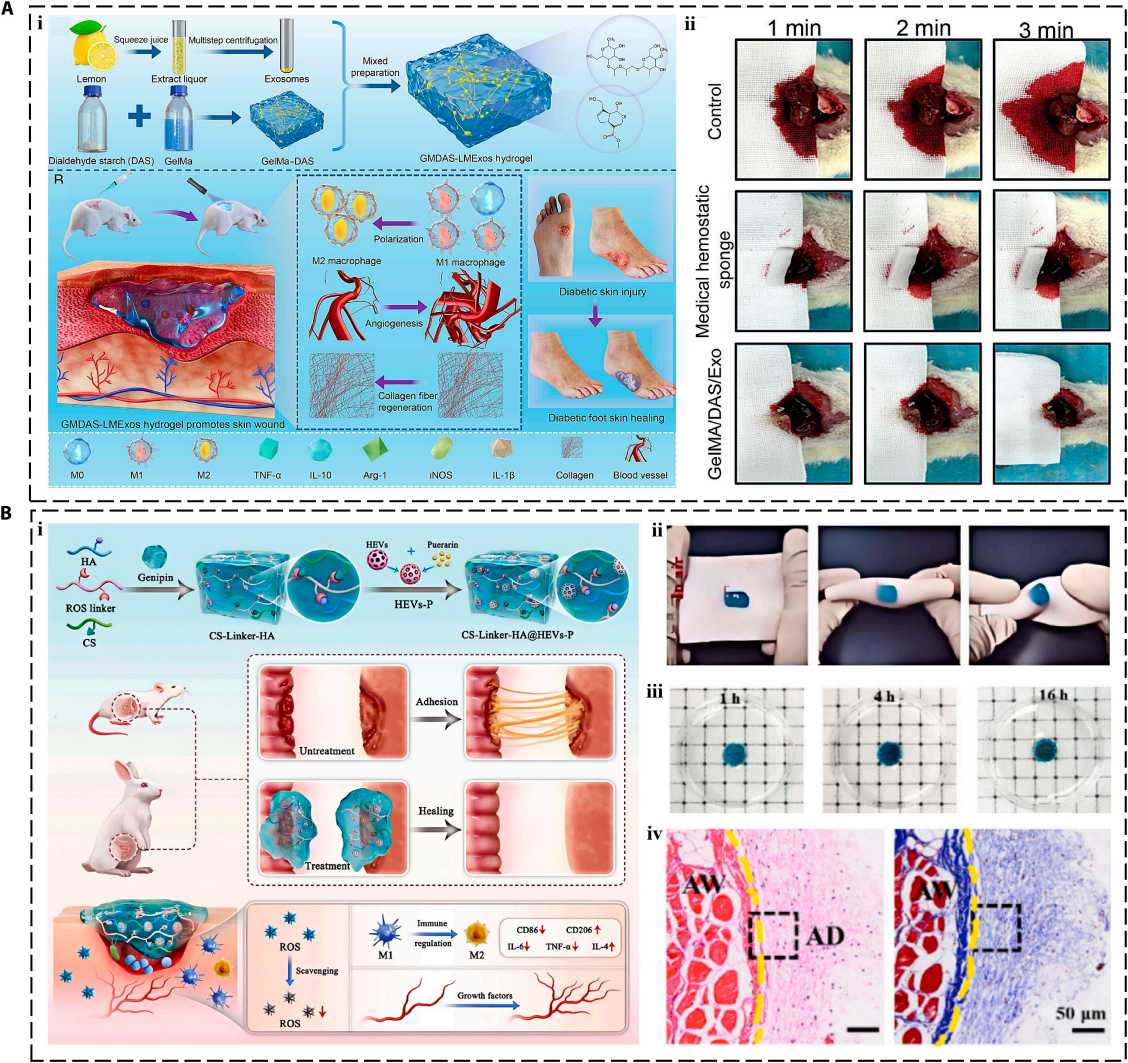
Combination of plant-derived exosomes (PLDEs) and hydrogels. (A) (i) Schematic of lemon-derived exosome-loaded hydrogel fabrication and its therapeutic mechanism. (ii) Comparison of hemostatic effect between GelMA/DAS/Exo hydrogel (lemon exosomes were loaded into a hydrogel constructed of gelatin methacryloyl and dialdehyde starch), medical hemostatic sponge, and control group. Adapted and reprinted with permission from Ref. [121]. Copyright 2025 The Author(s). Published by Springer Nature. (B) (i) Schematic of reactive oxygen species (ROS)-responsive hydrogel loaded with puerarin-engineered honeysuckle extracellular vesicles (HEVs-P). (ii) Demonstration of the adhesive performance of CS-Linker-HA@HEVs-P hydrogel (honeysuckle-derived extracellular vesicles engineered with puerarin were incorporated into the hydrogel developed by cross-linking chitosan and hyaluronic acid) under mechanical manipulation. (iii) Antiswelling test images. (iv) Representative hematoxylin and eosin and Masson’s trichrome staining of wound tissues. Adapted and reprinted with permission from Ref. [137]. Copyright 2025 Elsevier B.V.

Hydrogels are hydrophilic polymer networks capable of absorbing significant amounts of water while maintaining structural integrity. This unique property makes them an ideal platform for skin repair. Hydrogels resist infection, sequester exudates, and maintain a hydrated microenvironment to facilitate healing. Furthermore, their 3-dimensional (3D) matrix is adept at encapsulating, stabilizing, and controllably releasing bioactive molecules [118,119]. Hydrogels loaded with PLDEs significantly enhance their stability and enable sustained in situ release at the target site [120]. Common loading methods currently include physical adsorption, blended cross-linking, and in situ gelation. Physical adsorption exploits the hydrogel’s inherent hydration and adsorption capacity. In this process, the fully swollen hydrogel is deswollen using a solvent to remove bulk water and expose its porous architecture. The extracted EVs are then introduced and are passively adsorbed into the hydrogel matrix to form EV-loaded composites (Fig. 4A) [121]. In blended cross-linking, PLDEs are mixed with hydrogel precursor solutions (e.g., gelatin and HA). This process is followed by physical or chemical cross-linking (e.g., photopolymerization and ion cross-linking) to induce gelation. The PLDEs become encapsulated within the network [122]. Wang et al. [123] prepared a hydrogel by first incorporating adipose-derived mesenchymal stem cells exosomes into a solution containing thiolated HA, gelatin, and heparin, followed by cross-linking with PEG diacrylate. In in situ gelation, a sol–gel system containing PLDEs and polymers is injected into the target site via a dual-chamber syringe along with a cross-linking agent, forming a gel under physiological conditions. Zhao et al. [124] developed a hybrid wound dressing by integrating human umbilical vein endothelial cell (HUVEC)-derived EVs into a gelatin methacryloyl (GelMA) hydrogel matrix. They demonstrated that this dressing promotes tissue repair and enables sustained release of EVs.

Based on material sources, hydrogels used for PLDE delivery are primarily categorized as follows. Natural polymer-based hydrogels have inherent bionic properties, excellent biocompatibility, and good safety owing to their highly similar structure and composition to natural extracellular matrix. Given their favorable biodegradation kinetics and tunable cellular interactivity within the native tissue milieu, natural polymer-based hydrogels have been adopted as a premier platform for EV delivery. Hydrogels utilized for encapsulating EVs are chiefly formulated from natural biopolymers, which fall into 2 primary categories: polysaccharides (e.g., chitosan, alginate, and HA) and proteins (e.g., fibronectin, collagen, and gelatin) [125–128].

##### Chitosan

Chitosan, a biocompatible and biodegradable cationic polysaccharide derived from the deacetylation of chitin (found in arthropod exoskeletons), is widely used in the fabrication of functional hydrogels owing to its low toxicity, minimal immunogenicity, and intrinsic antibacterial activity [129]. Chitosan hydrogels possess a unique set of inherent properties, including antimicrobial activity, mucoadhesiveness, affinity for negatively charged cell membranes, and ease of functionalization. These attributes collectively contribute to their utility and enhanced therapeutic appeal as platforms for PLDE delivery [130]. Zhao et al. [131] encapsulated stem cell exosomes within chitosan hydrogels, significantly promoting full-thickness skin wound closure and reepithelialization. This approach presents a viable platform for the comprehensive repair of skin defects, as well as for applications in skin tissue engineering. Zhang et al. [132] developed a hydroxybutyl chitosan hydrogel loaded with BMSC-exosomes and found that it demonstrates potent activity in promoting tissue remodeling in a refractory skin wound model. However, the poor solubility of chitosan and the often inadequate temporal control of drug release from its hydrogels are notable drawbacks that collectively limit their translational progress.

##### Alginate

Alginate is a linear anionic polysaccharide copolymer consisting of (1→4)-linked β-d-mannuronate (M) and α-l-guluronate (G) residues and is sourced from brown algae and bacterial fermentation. Given their biocompatibility, cost effectiveness, and mild gelation conditions, alginate-based hydrogels are extensively utilized in tissue engineering, cell encapsulation, and controlled DDS [133]. Vipin et al. [134] developed a novel sprayable thermosensitive polysaccharide-based hydrogel loaded with exosomes via Schiff base reactions, incorporating aldehydes and amino groups in alginate. This hydrogel elicits a multifaceted therapeutic response in wound healing, markedly accelerating reepithelialization, enhancing granulation tissue formation and collagen deposition, promoting hair follicle neogenesis, mitigating inflammation, and stimulating robust neovascularization. To address hypoxic and inflammatory wound microenvironments, Zhang et al. [135] developed a sprayable alginate hydrogel coloaded with oxygen-releasing microspheres and exosomes. Its dual-action mechanism fosters M2 macrophage polarization and significantly improved healing outcomes, including expedited wound closure, efficient reepithelialization, organized collagen deposition, enhanced vascularization, and a resolved inflammatory response.

##### Hyaluronic acid

HA is a high-molecular-weight polysaccharide and a key glycosaminoglycan defined by its repeating disaccharide structure. It is native to all mammalian tissues, with the highest concentrations residing in the extracellular matrix of soft connective tissue [136]. HA hydrogels have been extensively fabricated into multifunctional carriers owing to their biocompatibility, nonimmunogenicity, tunable mechanics, and enzyme-mediated degradability (Fig. 4B) [137]. Utilizing various cross-linking methods, these carriers deliver therapeutic payloads such as MSCs, drugs, and growth factors, demonstrating efficacy in diverse regenerative and anti-inflammatory applications, including wound healing and vascular repair [117]. The published research confirms that Wang et al. [96] combined PLDEs derived from olive leaves with HA–TA hydrogels to form a composite system that effectively mitigates UV-induced skin damage and combats photoaging by down-regulating the NF-κB signaling pathway. Yang et al. [67] used a macroporous HA hydrogel fabricated via rapid lyophilization–thawing cycles to encapsulate human umbilical cord MSC-derived exosomes and coat with antimicrobial peptides. This construct orchestrates a proregenerative microenvironment by coordinately influencing fibroblast, endothelial, and macrophage behaviors and inhibiting fibrosis, culminating in enhanced tissue regeneration with reduced scarring in infected deep burns.

##### Other types

Protein-based hydrogels represent a major class of biomaterials for EV delivery. Among these, gelatin, derived from the denaturation of collagen, is widely used. Its methacryloyl-modified form (GelMA) marries favorable mechanical properties with inherent bioactivity, making it ideal for 3D EV culture and release. Stem cell EVs delivered via GelMA hydrogels orchestrate a proregenerative response in cartilage repair, enhancing chondrocyte and BMSC proliferation/migration, promoting anabolism while inhibiting catabolism, and facilitating chondrogenesis [126]. Fibrin, a natural hemostatic polymer derived from blood, exhibits low immunogenicity and favorable biocompatibility, making it valuable as a tissue sealant and engineering scaffold [138]. Some novel materials that have demonstrated superiority in nerve repair treatments include a poly(3,4-ethylenedioxythiophene)-integrated fish swim bladder, a type of magnetic nanochain-induced anisotropic nerve assembly for spinal cord injury repair, and a type of biomimetic hydrogel microfibers [139–141]. Exosomes also exhibit advantages in nerve repair. He et al. [142] demonstrated that encapsulating EVs within fibrin gel (“gel-exosomes”) confers novel therapeutic functionalities. In a spinal cord injury model, this construct improves behavioral and electrophysiological outcomes, up-regulates neural markers at the lesion site indicating proneurogenic effects, and thus holds considerable translational promise as a biocompatible strategy for neural repair. Lin et al. [143] developed a bioprinted fabric based on the decellularized extracellular matrix of fish skin and found that this material can effectively promote cell adhesion and proliferation. Given its porous structure and high specific surface area on the hydrogel framework, the decellularized extracellular matrix hydrogel can load various active molecules, such as exosomes, thereby further enhancing the healing effect of wounds.

In contrast to natural polymers, synthetic polymer-based hydrogels offer precise control over key structural parameters, including molecular weight, block copolymer architecture, degradable linkage chemistry, and cross-linking network topology. This property enables the production of custom-sized, tunable structures with controlled degradation rates. Furthermore, synthetic hydrogels exhibit high reproducibility [120]. These hydrogels are primarily composed of cross-linked synthetic hydrophilic polymers and are used for delivering EVs. Examples of synthetic hydrogels include PEG, poly(acrylic acid) derivatives, poly(lactic-*co*-glycolic acid), poly(vinyl alcohol), and poly(2-methylacrylates-*co*-2-hydroxyethyl methacrylate). Kwak et al. [144] used injectable, biodegradable PEG controlled release reservoirs as extracellular microvesicles. By modulating the degree of cross-linking and the resultant network mesh size of the hydrogel, they achieved tunable degradation timelines (6 to 27 d) to release M2-EVs, thereby maximizing therapeutic efficacy for skin wound healing.

#### Microneedles

**Fig. 5.**
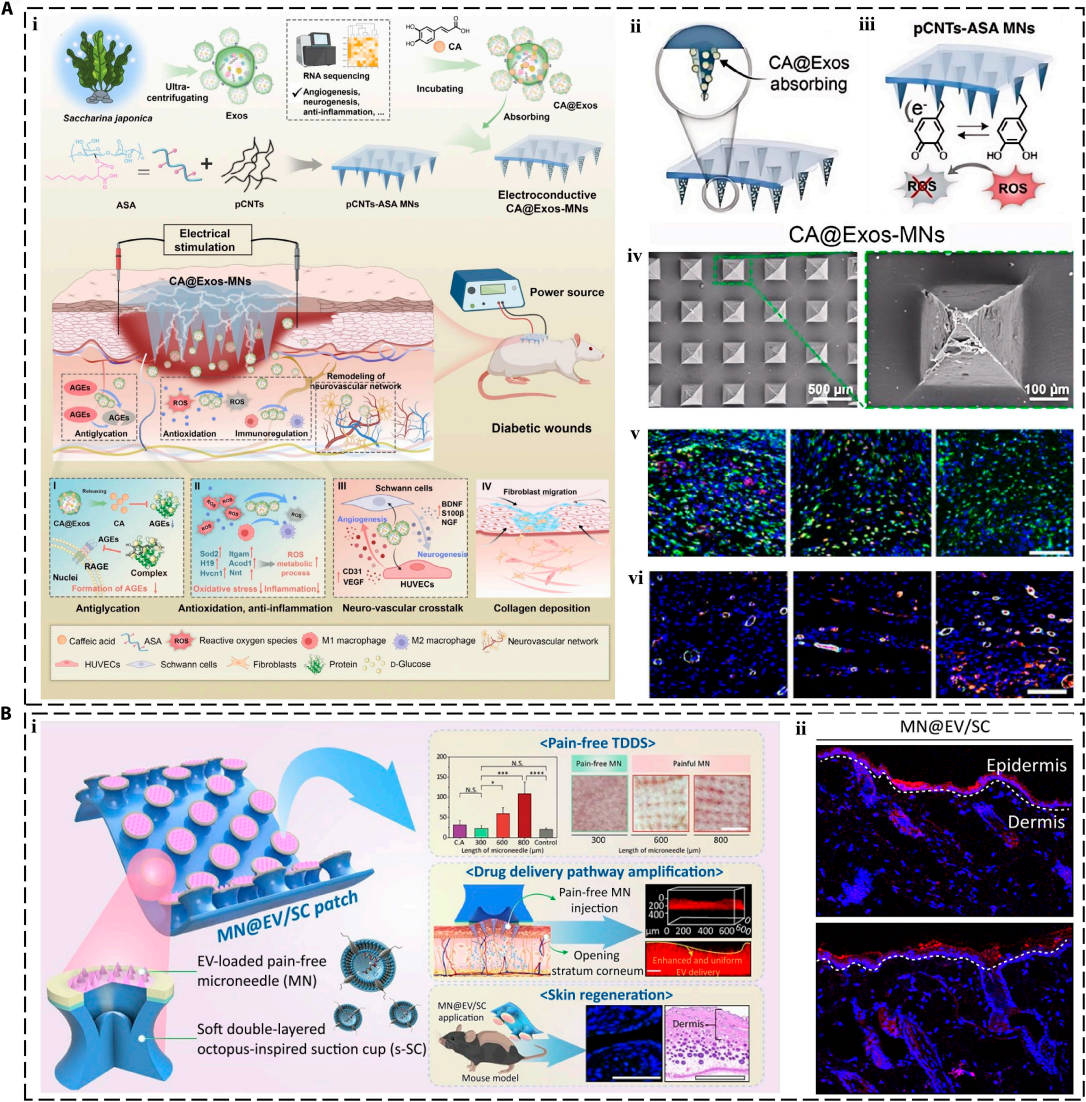
Combination of plant-derived exosomes (PLDEs) and microneedles. (A) (i) Schematic of conductive microneedles functionalized with *Saccharina japonica*-derived exosomes. (ii and iii) Schematic of the fabrication and therapeutic mechanism of exosome (Exo)-loaded microneedles. (iv) Scanning electron microscopy characterization of *S. japonica* exosome-derived biological signals with electroconductive microneedles (CA@Exos-MNs). (v) Immunofluorescence analysis of CD68-positive macrophages in wound tissues. Scale bar, 100 μm. (vi) Immunofluorescence assessment of CD31 expression. Scale bar, 100 μm. Adapted and reprinted with permission from Ref. [147]. Copyright 2025 The Author(s). Advanced Science published by Wiley-VCH GmbH. (B) (i) Schematic of the microneedles engineered with extracellular vesicles (MN@EV/SC) transdermal patch. Data are expressed as the means ± SD. N.S. (not significant), *P < 0.05, **P < 0.01, ***P < 0.005, and ****P < 0.001 by one-way ANOVA with Tukey’s post hoc test were considered. (ii) Fluorescence analysis of skin cryosections. Adapted and reprinted with permission from Ref. [153]. Copyright 2025 The Author(s). Published by Springer Nature. N.S., not significant.

Microneedle (MN) patches are minimally invasive transdermal platforms composed of microscale needle arrays that painlessly penetrate the stratum corneum to deliver therapeutics. They exhibit excellent safety profiles owing to their biocompatible materials and minimally invasive nature, which reduce risks of infection, bleeding, and patient discomfort while ensuring high compliance [145]. Their growing prominence in biomedicine is due to this patient-compliant mode of administration. For delivering therapeutic EVs, MNs provide a potent alternative to injections by enabling localized, controlled release at target sites to achieve enhanced therapeutic outcomes (Fig. 5A) [146,147]. Owing to the skin’s efficient barrier function, traditional hydrogels are largely restricted to protecting the wound bed and enabling sustained drug release at the surface, with a limited capacity to reach deeper tissue layers [148]. A strategic solution to this challenge uses MNs [149]. Common materials used for preparing MNs include gelatin, poly(lactic-*co*-glycolic acid), poly(vinyl alcohol), and chitosan. MNs are utilized to deliver EVs and EV-derived molecules [150]. The core–shell architecture of the network enables spatiotemporal control over the release of bioactive cargo, achieving deep penetration, sustained release, and sequential delivery phases that collectively orchestrate a synergistic healing response [148]. The mechanistic basis of MN–EV integration relies on the physical encapsulation or affinity-based immobilization of EVs within the MN matrix; upon skin insertion, rapid dissolution or swelling of the needle matrix releases the EVs directly into the dermal and epidermal layers, where they can interact with resident cells to exert their therapeutic effects. MNs loaded with PLDEs typically use direct loading, wherein PLDEs are mixed with the hydrogel precursor solution and then filled into MN molds. After cross-linking, curing, and drying, MNs are formed. More complex hierarchical construction methods can achieve staged drug release [149]. Yang et al. [151] demonstrated for the first time that hair keratin MNs significantly promote hair growth in a mouse model by delivering activated exosomes. Shi et al. [152] fabricated a removable MN patch from chitosan lactate to deliver adipose-derived stem cell EVs. This system promotes cell proliferation by activating Wnt signaling; moreover, chitosan lactate (CL) and EVs synergistically accelerate hair regrowth through follicular cycle regulation. In addition, EVs delivered via MNs show great potential for treating skin wound healing (Fig. 5B) [153]. For instance, Yuan et al. [150] developed a GelMA-based MN patch to achieve controlled transdermal codelivery of EVs and etanercept. By promoting cell migration and angiogenesis, this strategy results in complete skin wound healing in diabetic mice, highlighting the promise of EV-laden MNs.

#### Nanoparticle/microsphere

Biomaterials based on microspheres are used as injectable microscaffolds, offering the advantage of minimally invasive procedures for therapeutic applications in various EV delivery systems. Meng et al. [154] proposed a “drug-excipient integration” design strategy, using freeze–thaw technology to rapidly prepare traditional Chinese medicine nanospheres *Aster* super self-assemblies (ATSSA) from *Aster tataricus* decoction. They then isolated PLDEs via gradient centrifugation and obtained engineered exosomes—EVs at nintedanib (EV@NIN)—through ultrasonic mixing and incubation. In this system, the interaction mechanisms between nanoparticles and exosomes, primarily electrostatic interactions, hydrogen bonding, and hydrophobic forces, play a critical role in determining the loading efficiency and release kinetics of PLDEs. These noncovalent forces facilitate stable association while allowing controlled dissociation under physiological conditions, thereby influencing the spatiotemporal release profile of therapeutic EVs. Furthermore, the nanospheres exhibit favorable safety profiles owing to their biocompatible composition (derived from natural herbal decoction) and the mild freeze–thaw preparation process, which minimizes cytotoxicity and systemic toxicity. These 3 carriers are delivered to the lungs via interstitial injection, bypassing hepatic first-pass metabolism and enhancing drug bioavailability. The compounds ATSSA, EV, and EV@NIN exert antifibrotic effects in idiopathic pulmonary fibrosis via multipronged mechanisms, such as modulating cytokine storms, rebalancing redox homeostasis, and inhibiting transforming growth factor-β1 (TGF-β1)-driven epithelial–mesenchymal transition and fibroblast-to-myofibroblast transition. This action is associated with a marked reduction in epithelial–mesenchymal transition and ECM deposition, indicating that suppression of myofibroblast activation and collagen synthesis underlies their therapeutic efficacy.

#### Topical injection

Injectable hydrogels are among the most prevalent biomaterial scaffolds for tissue engineering. Their utility extends beyond the treatment of superficial wounds to encompass the repair and regeneration of deep tissues and organs [155]. Hydrogels serve as versatile carriers for therapeutic payloads (e.g., drugs, growth factors, and cells), which can be injected directly into lesion sites. This minimally invasive strategy facilitates effective repair while avoiding complex surgeries. Notably, the local injection of EV-laden hydrogels, the most prevalent delivery method, enables spatiotemporally controlled release to optimize tissue regeneration [155]. This injectable system combines shear-thinning properties for easy delivery with in situ gelation capability upon injection, allowing for precise placement and forming a stable, cross-linked network locally [25]. In situ gelation can be accomplished through 2 principal strategies: direct coinjection of EVs, polymers, and cross-linkers or exploiting physiological stimuli such as ionic concentration, temperature, and pH to trigger gel formation. As a representative application, Cao et al. [156] delivered an injectable hydrogel loaded with human urine-derived stem cell exosomes via intrathecal injection, which effectively promotes angiogenesis and facilitates repair in a spinal cord injury model.

#### 3D bioprinting with bioinks

As an advanced fabrication method, 3D bioprinting converts digital models into intricate, customized 3D geometries by depositing bio-inks layer-wise, offering unprecedented control over scaffold architecture [157]. Bioink, an ink suitable for 3D printers, commonly incorporates hydrogels as a key component. 3D bioprinting offers precise control over scaffold architecture, porosity, and mechanical properties, enabling the fabrication of constructs engineered to accommodate the coloading of cells and EVs [158]. Born et al. [159] demonstrated the feasibility of 3D bioprinting in preparing viable exosome-laden constructs, showing that GelMA hydrogels encapsulating MSC-derived exosomes maintain their functional potency after printing and photopolymerization without compromising bioactivity. The release kinetics of exosomes can be precisely controlled by optimizing hydrogel cross-linking parameters. Beyond fabrication versatility, 3D bioprinted EV-laden scaffolds exhibit favorable safety profiles because they allow the local activity of exosomes while avoiding off-target effects, systemic toxicity, and the immunogenic and tumorigenic concerns associated with whole-cell therapies.

## Primary Applications and Mechanisms in Cosmetic Surgery

### Antiaging

Skin aging is a multifaceted process driven by intrinsic (genetic and metabolic) and extrinsic (e.g., UV radiation and pollution) factors [160]. The core mechanism of skin aging involves oxidative stress triggering DNA damage and inflammatory cascades, increasing matrix metalloproteinase (MMP) expression, and promoting collagen and elastin degradation [161]. Current mainstream treatment options include hydrogel therapy, growth factor therapy, and traditional methods. In hydrogel therapy, highly hydrated materials are utilized for controlled drug release, reducing UV damage and promoting skin repair. In growth factor therapy, human fibroblast-conditioned medium enhances collagen synthesis and skin regeneration by secreting growth factors and bioactive molecules. Traditional methods such as topical antioxidants and laser therapy are also used, but they are limited by poor penetration and potential side effects [45,162,163].

PLDEs are garnering increasing attention in antiaging research as an attractive platform largely owing to their biomimetic lipid bilayer structure, excellent biocompatibility, low cytotoxicity, and combined antioxidant, anti-inflammatory, and regenerative properties [164–166]. Research has proven that vesicles isolated from *Ecklonia cava* plants significantly mitigate oxidative stress and photoaging damage by up-regulating heat shock protein 70 (HSP70) expression and down-regulating inflammatory signaling pathways including TNF-α, MAPK, and NF-κB [167]. Exosomes derived from the medicinal mushroom *Escherlinus linteus* exert beneficial effects against UV-induced aging. Kale can inhibit skin aging symptoms and increase the expression of type I collagen and antioxidant enzymes in skin tissue, demonstrating significant potential in improving skin aging [168]. PLDEs transport a wide array of phytochemicals, demonstrating considerable chemical diversity. Notable examples encompass antioxidants such as ascorbic acid, catalase, and glutathione from citrus; vitamin C from strawberry exosomes; phenolic and flavonoid compounds prevalent in citrus fruits; and specific bioactive molecules such as gingerol, cannabidiol, trans-δ-glucoside, anthraquinones, and curcumin with phenolic acids [169–175]. Pomegranate fruit is a source of identified functional antioxidants such as 10,16-dihydroxypalmitic acid and *O*-methylated ellagic acids, implicating them in potential cutaneous antiaging applications [176]. Lavender-derived ELNs attenuate UVB-induced skin inflammation and aging through miR166-mediated regulation of inflammation and collagen. These metabolites exhibit significant biological effects in vesicle-targeted cells, effectively neutralizing reactive oxygen species (ROS), ameliorating oxidative damage to key skin cells (e.g., fibroblasts and keratinocytes), and thereby delaying the progression of photoaging and chronological aging [177]. In addition, the down-regulation of Smad7 expression by specific miRNAs from kale-derived exosomes provides a mechanistic basis for their observed stimulation of collagen synthesis [178]. Li et al. [179] demonstrated that lavender-derived exosomes, through miR166-mediated inflammation and collagen regulation, attenuate UVB-induced photoaging.

Current interventions against photoaging primarily emphasize preventive sun protection and superficial repair but remain limited in scope. Consequently, identifying bioactive molecules that can actively counteract UV damage is crucial for developing advanced pharmaceuticals and cosmeceuticals. In this context, research demonstrates that the OLELNVs@HA/TA hydrogel, a composite of olive-leaf-derived EVs (OLELNVs), HA, and TA, effectively mitigates UV-induced skin damage while concurrently promoting tissue regeneration and repair [96]. Fungal exosome-like nanovesicles function by delivering miR-CM1, which cross-regulates gene expression in human keratinocytes to counteract photoaging. This process involves suppressing Mical2 and MMP1 while enhancing collagen type I alpha 2 chain (COL1A2); rebalancing oxidative stress markers (reducing ROS/malondialdehyde, elevating superoxide dismutase [SOD]); and decreasing cellular senescence (senescence-associated β-galactosidase), thereby attenuating UV-induced skin aging [62]. Ginseng-root-derived exosomes confer protection against UV radiation and oxidative stress in human dermal fibroblasts and melanocytes. This effect is achieved by inhibiting the activation protein 1 signaling pathway and reducing ROS production, which collectively mitigates the UV-induced senescent phenotype [180]. Exosomes derived from shiitake mushrooms comprise a diverse cargo of RNAs, proteins, lipids, polyphenols, and flavonoids, imparting notable antioxidant and radioprotective capacities. This profile suggests their prospective utility in mitigating tissue damage induced by infrared radiation [181]. Aloe vera exosomes elicit significant antiphotoaging and antioxidant effects by activating the Nrf2/antioxidant response element (ARE) pathway, thereby reducing UV-induced DNA damage while maintaining high biocompatibility [177]. In summary, compared with traditional therapeutic approaches and animal-derived exosomes, PLDEs exhibit lower immunogenicity, higher safety, and scalability for industrial-scale manufacturing, a critical attribute for their clinical and commercial translation in antiaging applications.

### Anti-inflammatory

Cutaneous injury from various causes (e.g., trauma, burns, and surgery) triggers a conserved reparative program involving 4 interrelated phases: hemostatic, inflammatory, proliferative, and remodeling [182,183]. The inflammatory phase, the second critical stage of wound healing, is essential for clearing pathogens and debris through the recruitment of neutrophils, macrophages, and lymphocytes, thereby initiating subsequent repair. Although conventional anti-inflammatories such as glucocorticoids are effective, their prolonged use is associated with adverse effects including skin atrophy. Therefore, developing natural anti-inflammatory agents that combine high efficacy with safety has become a key research focus [184]. Recent studies have confirmed that PLDEs possess significant anti-inflammatory potential. Curcumin is the most important chemical component in turmeric, capable of exerting pharmacological effects. Turmeric-derived exosomes contain high levels of curcumin and exert dual anti-inflammatory and antioxidant effects by concurrently suppressing proinflammatory cytokines (TNF-α, IL-6, and IL-1β) and enhancing heme oxygenase-1 (HO-1) activity [33]. Following internalization by skin tissues, coriander-derived exosome (CDE)-like nanovesicles promote cellular migration. They mitigate inflammation and accelerate wound healing by up-regulating endogenous antioxidant enzymes to scavenge ROS [185]. *Lithospermum erythrorhizon* wound-healing tissue-derived exosome-like vesicles inhibit TNF-α production in a dose-dependent manner in LPS-induced inflammatory models, confirming their significant anti-inflammatory properties [186]. Papaya PLDEs are rich in anti-inflammatory compounds, such as the flavonoid 2,3-dihydro-3,5-dihydroxy-6-methyl-4*H*-pyran-4-one (DDMP). At 17.5 μM, DDMP demonstrates excellent radical scavenging activity (81.1%) along with anti-inflammatory, antibacterial, and antifungal properties [187]. Other anti-inflammatory molecules in papaya PLDEs include *N*-hexadecanoic acid, which functions as a phospholipase A_2_ [188]; 5-hydroxymethylfurfural, a multipathway inhibitor that concurrently targets the MAPK, NF-κB, and Akt/mammalian target of rapamycin signaling cascades to suppress inflammation [189]; sitosterol, which reduces the IL-13-induced expression of neurokinin receptor 1-related in BEAS-2B cells [190]; and 9,12-octadecadienoic acid, 11-octadecenoic acid, 10,13-octadecadienoic acid [191], 2-oxopropionamide, and piperazine, which exert anti-inflammatory effects by functioning as antagonists of histamine and serotonin receptors [192]. Macrophages can classified into proinflammatory M1 and proresolving M2 phenotypes. Goldberry-derived ELNs exert anti-inflammatory effects by suppressing M1-associated mediators and promoting a shift toward the reparative M2 polarization state [193]. Blackberry-derived ELNs mediate their anti-inflammatory effect by inhibiting IL-6 secretion in LPS-activated RAW264.7 macrophages [194]. Strawberry-tree-derived ELNs exert a concerted anti-inflammatory activity in LPS-stimulated RAW264.7 cells. This activity is characterized by the concomitant down-regulation of key inflammatory mediators and signaling molecules at the mRNA and protein levels [195]. Collectively, these findings position PLDEs as a promising multitargeted strategy for managing cutaneous inflammation. They effectively modulate key inflammatory pathways while avoiding the adverse effects commonly associated with conventional therapies.

### Skin whitening and spot reduction

The darkness or lightness of human skin tone is primarily determined by the amount of melanin in the skin. Melanin is biosynthesized within melanocytes through a cascade of enzymatic reactions, with tyrosinase serving as the rate-limiting catalyst. Its core function lies in absorbing UV radiation and scavenging free radicals, thereby protecting cellular DNA from UV damage, maintaining skin barrier function, and reducing the risk of skin diseases [196–200]. However, melanin production leads to darker skin tones, and its uneven deposition may cause hyperpigmentation and dark spots. This phenomenon drives sustained market demand for safe and effective skin-lightening and spot-reducing products [201]. Currently, numerous natural or synthetic whitening ingredients are widely used in skincare products and cosmetics to inhibit melanin production and improve uneven skin tone. The action targets of these ingredients often focus on inhibiting the activity of tyrosinase, the enzyme that acts as the rate-limiting step in the melanin synthesis pathway (tyrosine → dopaquinone → melanin). Reducing its activity directly decreases melanin production. However, many traditional whitening ingredients, such as certain synthetic inhibitors or high-concentration natural extracts, may present limitations, including skin irritation, long-term safety concerns, or low transdermal absorption efficiency [202]. PLDEs exhibit considerable promise as novel agents for skin-lightening applications owing to their excellent biocompatibility and efficient delivery capabilities. PLDEs exert synergistic whitening effects through multiple mechanisms. One is by directly regulating melanin synthesis pathways. Exosomes derived from the symbiotic bacteria of Japanese camellia (*Camellia japonica*) and from the leaves and stems of Chinese sumac (*Rhus verniciflua* Stokes) and tea leaves significantly down-regulate the expression of key melanin synthesis genes and tyrosinase activity, thereby reducing melanin content. In addition, camellia exosomes can indirectly down-regulate MITF expression by inhibiting the phosphorylation of signaling pathways such as MAPK, Akt, and extracellular signal–regulated kinase, thereby regulating melanin production at a more fundamental level [203–205]. Another one is by exerting antioxidant and anti-inflammatory activities. PLDEs possess potent antioxidant and anti-inflammatory activities, conferred by their intrinsic components or delivered phytochemicals (e.g., phenols and flavonoids), which scavenge free radicals and protect melanocytes from oxidative damage. As a specific example, exosomes derived from *Camellia sinensis* (DB-21 exosomes) notably suppress the expression of key proinflammatory cytokines and mediators (inducible nitric oxide synthase, cyclooxygenase-2, and prostaglandin E_2_) [206]. Reducing inflammation diminishes its activating effect on melanocytes, thereby indirectly inhibiting pigmentation. The anti-inflammatory and antioxidant properties of PLDEs synergize with their direct melanogenesis-suppressing capabilities, collectively promoting skin tone brightening and dark spot reduction [203,207]. Last is by promoting skin repair. Certain PLDEs (such as those derived from yam beans) enhance skin regeneration and improve hyperpigmentation and melasma [208]. Compared with traditional whitening ingredients and animal-derived exosomes, PLDEs offer distinct advantages. First, as natural nanocarriers, PLDEs effectively deliver their endogenous bioactive substances (such as miRNAs, phenols, and flavonoids) to target skin cells, enhancing bioavailability. Second, PLDEs simultaneously target multiple stages of melanin synthesis (enzyme inhibition, gene regulation, and signaling pathway intervention) while exhibiting antioxidant and anti-inflammatory properties, achieving synergistic whitening effects. In addition, derived from edible plants, PLDEs typically offer superior biocompatibility and lower potential irritation. They are widely available and sustainable. Finally, PLDEs can be mass produced through methods such as plant cell culture. In summary, PLDEs suppress melanin production via multiple mechanisms, reduce oxidative stress, counteract inflammatory pathways, and promote skin repair. They offer a novel approach and highly promising natural solution for safely and effectively addressing uneven skin tone and pigmentation issues [203,204,206,208].

### Skin barrier repair

The skin barrier, primarily composed of stratum corneum, sebum film, and tight junctions, serves as the body’s first line of defense for maintaining health by warding off external irritants and preventing moisture loss. Once the skin barrier is compromised, it often manifests as dryness, sensitivity, and inflammation and may even trigger conditions such as atopic dermatitis and rosacea. Conventional wound care methods such as dressings have several limitations, including poor antimicrobial efficacy, insufficient mechanical strength, and inadequate moisture retention, making wound healing difficult to comprehensively promote [209]. PLDEs offer a novel solution as natural nanocarriers. These vesicles are rich in anti-inflammatory and antioxidant bioactive compounds, such as polyphenols and flavonoids, from source plants, along with functional miRNAs, thereby enabling the precise regulation of key pathways in skin repair. Lavender-derived exosomes confer dual protection by neutralizing ROS and down-regulating the expression of inflammatory mediators, thereby alleviating oxidative stress and inflammation. miR-166 family members, such as cpa-miR-166e and zma-miR-166h-3p, target and regulate the Wnt/β-catenin and MAPK signaling pathways, promoting collagen synthesis and epidermal cell proliferation, thereby accelerating reepithelialization [179,209]. Red-onion-derived nanovesicles orchestrate barrier repair through a concerted action: They mitigate oxidative stress by scavenging ROS and boosting SOD activity while simultaneously suppressing proinflammatory cytokines, thereby attenuating skin inflammation and facilitating tissue restoration [210]. By regulating key genes such as collagen I and elastin, plant exosomes accelerate the migration and proliferation of keratinocytes and fibroblasts, optimize wound reepithelialization and collagen deposition, and restore skin barrier integrity. They also suppress T cell hyperactivation (e.g., by reducing IL-17A expression) and promote angiogenesis. Furthermore, plant exosomes enhance skin microcirculation and function by delivering miRNAs (e.g., miR-192-5p) that target genes such as desmocollin 1 (DSC1). Through their bioactive components, PLDEs orchestrate antioxidant, DNA repair, and antimelanogenic effects by modulating key signaling hubs, including the Nrf2/ARE, MAPK/activation protein 1, phosphatidylinositol 3-kinase (PI3K)/Akt, and MITF/tyrosinase pathways, positioning them as versatile agents for preventing and treating UV-induced photodamage [211]. Compared with traditional drugs, PLDEs demonstrate more comprehensive barrier repair efficacy through their multitarget regulatory capabilities (simultaneously intervening in inflammation, oxidative stress, cell proliferation, and ECM remodeling). These characteristics position PLDEs as a promising tool to overcome existing therapeutic limitations.

### Wound healing

Wound healing is a complex, multiphase physiological response that is orchestrated through 4 sequential yet interdependent stages: hemostasis, inflammation, proliferation, and tissue remodeling. Impairment at any stage may culminate in aberrant healing, manifesting as persistent wounds, delayed closure, or pathological scar hyperplasia. Current clinical wound management strategies primarily include traditional dressings, growth factor gels, and synthetic scaffolds. Although these approaches play a role in protecting the wound surface and maintaining a moist environment, they generally suffer from limitations such as single functionality, susceptibility to inactivation of active ingredients, and limited capacity to regulate the healing microenvironment. Their efficacy is particularly unsatisfactory in complex wounds complicated by systemic diseases such as diabetes. Owing to their inherent biocompatibility and role as natural carriers, plant exosomes are considered a versatile delivery platform in regenerative medicine. They transport a diverse array of signaling molecules, including miRNAs, proteins, and lipids, to precisely regulate target cell activities [212]. The inflammatory response in the early wound healing phase serves the critical physiological function of pathogen clearance and tissue debridement, but prolonged or excessive inflammation impedes healing. Recent studies have confirmed that various PLDEs possess potent anti-inflammatory capabilities. Plant-derived EVs promote skin wound repair by modulating pathological ROS/reactive nitrogen species levels back to homeostatic levels, thus counteracting the oxidative stress that delays healing and fosters chronic wounds [212,213]. CDEs confer enhanced oxidative stress tolerance and promote keratinocyte migration by activating the Nrf2 signaling pathway and up-regulating antioxidant enzymes such as HO-1. When encapsulated within a hydrogel for sustained delivery, CDEs significantly accelerate wound closure and epithelial regeneration in a murine full-thickness skin defect model [185]. Dendrobium-derived exosome-like nanovesicles mediate wound healing by down-regulating IL-1β expression, thereby attenuating inflammation and accelerating repair in murine wounds [214]. The synergistic design of a lemon exosome/GelMA/dialdehyde starch (DAS) hydrogel accelerates diabetic wound repair by reprogramming the immune milieu. It shifts macrophages toward the prohealing M2 phenotype, consequently boosting HUVEC-mediated angiogenesis and fibroblast activity to facilitate tissue regeneration [121]. Regarding angiogenesis, multiple studies have confirmed that PLDEs possess distinct proangiogenic potential. For instance, grapefruit exosomes enhance the angiogenic capacity of HUVECs by regulating vascular endothelial growth factor (VEGF)-related pathways [215]. PLDEs derived from aloe vera and ginseng also synergistically promote the establishment of new vascular networks by modulating the angiopoietin-1/Tie2 signaling pathway, thereby providing nutritional and oxygen support to wounds [216,217]. Furthermore, wheatgrass exosomes at a concentration of 200 μg/ml not only significantly enhance the expression of type I collagen mRNA but also promote the migration of endothelial and fibroblast cells by activating the integrin–focal adhesion kinase (FAK) signaling axis, demonstrating superior reepithelialization and matrix remodeling capabilities [218]. In terms of immune regulation and anti-infection, dandelion exosomes effectively reduce bacterial load and inflammatory response at wound sites by directly binding to and neutralizing staphylococcal exotoxins [219]. PLDEs derived from turmeric and edible kudzu root can regulate the Toll-like receptor 4 (TLR4)/NF-κB pathway, inducing macrophage M2 polarization to synergistically suppress excessive inflammation and accelerate repair [56,70]. Compared with animal-derived exosomes, PLDEs exhibit lower pathogenic risks and superior biosafety. Moreover, their natural plant metabolite composition endows them with intrinsic antioxidant and anti-inflammatory activity. Through further integration with advanced biomaterials such as hydrogels and MNs, PLDEs can achieve prolonged local retention and controlled release, offering a highly promising natural nanotherapeutic strategy for the clinical management of complex wounds and hard-to-heal ulcers.

### Scar treatment

Pathological scars result from fibroblast overactivation, abnormal deposition of extracellular matrix, and disrupted collagen metabolism during wound healing, substantially impairing both appearance and function [220,221]. Current clinical interventions, such as steroid injections, laser therapy, and surgery, have prolonged treatment cycles, high recurrence rates, or associated tissue atrophy, making it difficult to achieve deep-level regulation of scar formation [222,223]. Owing to their natural origin, favorable safety profile, and capacity for multifaceted regulation, PLDEs have gained considerable interest as a promising candidate for preventing and treating pathological scarring. However, research on the role of PLDEs in scar management remains limited; most evidence has been derived from nonspecific studies involving animal EVs or plant extracts. In preclinical studies, rose stem cell exosomes improve the texture and contracture of scars resulting from trauma and acne, enhance tissue repair quality and aesthetic outcomes, accelerate healing and reduce inflammation in acute wounds, and improve the long-term scar growth quality in chronic wounds [164,224]. Betaine-derived exosomes regulate the balanced expression of type I/III collagen and hyaluronan synthase-2 in fibroblasts while inhibiting their migratory capacity, suggesting their potential in regulating ECM homeostasis [225]. Although direct evidence remains to be strengthened, the anti-inflammatory, antioxidant, and signal-modulating properties of PLDEs provide a theoretical basis for their potential intervention in scar formation. Potential mechanisms include regulating TGF-β/Smad and MAPK pathways through plant miRNAs or bioactive metabolites to suppress abnormal activation and transdifferentiation of fibroblasts, thereby simultaneously improving the healing microenvironment by alleviating local oxidative stress and chronic inflammation, and inhibiting excessive fibrosis [226]. Excessive mitochondrial fission is observed in macrophages from human and mouse hypertrophic scar tissues, and this imbalance is controlled by Aurora kinase B-mediated Ser616 site dynamin-related protein 1 phosphorylation. Li et al. [227] used artificial intelligence machine learning combined with biological validation to identify natural small molecule asiatic glycosides (ASs) as a potent Aurora kinase B inhibitor and developed a loaded AS (AS@cRGD-EVs). The cyclic arginine–glycine–aspartic acid (cRGD) decoration of EVs enables AS to be targeted and delivered to macrophages in wound tissue, exhibiting significant anti scar effects. Future investigations should systematically characterize the impact of PLDEs from diverse botanical sources on scar formation, spanning in vitro cellular responses, in vivo animal models, and, ultimately, human clinical scar tissues, while exploring their combined application with existing therapies or biomaterials. Although in the exploratory phase, PLDEs offer considerable promise as a natural platform combining biological functions with nanocarrier properties for developing novel and integrated therapeutic strategies against pathological scarring.

### Hair loss prevention and hair follicle regeneration

Hair loss is a common dermatological concern that encompasses several primary forms, including androgenetic alopecia, alopecia areata, and telogen effluvium [228]. Its pathogenesis involves multiple factors, including genetic predisposition, hormonal imbalance, abnormal hair follicle microenvironment, and local inflammatory responses. Current clinical treatment options include minoxidil and finasteride and hair transplantation surgery [229,230]. However, medication requires long-term maintenance and carries potential side effects, while surgical methods are invasive, costly, and limited by donor availability. Therefore, safer, more effective, and sustainable therapies are needed. In recent years, PLDEs have shown considerable promise in hair follicle regeneration, offering a novel approach in the context of alopecia therapy owing to their excellent skin permeability, low immunogenicity, and multiple biological functions. PLDEs synergistically promote hair regrowth through multiple pathways, including regulating hair follicle cycle-related signaling pathways, alleviating local inflammation, and counteracting oxidative stress. For example, papaya-derived exosomes substantially inhibit LPS-induced nitric oxide synthesis and induce a favorable shift in the cytokine milieu of RAW264.7 macrophages, characterized by reduced levels of IL-1β and IL-6 alongside increased IL-10 expression. This finding suggests their capacity to regulate the immune microenvironment surrounding hair follicles [187]. Shiitake mushroom exosomes confer protection against inflammation-mediated hair follicle damage by suppressing the NLRP3 inflammasome, leading to the down-regulation of IL-6 and IL-1β at the transcriptional and protein levels [231]. A clinical study demonstrated that 5 treatments with plant-derived EV complexes within 5 months can significantly increase the density of hair visible to the naked eye in a 48-year-old male patient with Norwood grade 5 androgenic alopecia, suggesting the potential benefit of PLDEs in treating refractory hair loss. Another pilot study with 20 patients receiving exosome treatment also showed significantly increased hair density from 105.4 to 122.7 counts/cm^2^ (*P* < 0.001) and significantly increased mean hair thickness from 57.5 to 64.0 mm (*P* < 0.001) after 12 weeks of treatment [232].Furthermore, a randomized controlled trial further confirmed that PLDE formulations containing extracts of *E. cava* and eastern cypress considerably promote hair regrowth in patients with androgenetic alopecia, with no adverse reactions reported, demonstrating their favorable clinical safety and tolerability [233]. At the mechanistic level, exosomes derived from germinated hemp seeds (E40) effectively counteract dihydrotestosterone-induced hair follicle damage, exhibiting superior protective effects to crude extracts. This result suggests their high bioavailability and therapeutic potential [234]. German iris rhizome exosomes enhance dermal papilla cell function by activating the Wnt/β-catenin signaling pathway, thereby promoting hair follicle regeneration and cycle restart [235]. Compared with traditional treatment approaches, PLDEs exhibit multitargeted, holistic regulatory properties, simultaneously acting on hair follicle cells, immune cells, and the vascular microenvironment. These findings indicate significant therapeutic potential in the context of hair follicle regeneration. The therapeutic mechanisms and applications of common plant exosomes in aesthetic medicine are summarized in Table 1.

**Table 1.** Therapeutic mechanisms and applications of PLDEs in aesthetic medicine

Biological activity	Plant source	Mechanism of action	Application	Ref.
Antiaging and skin rejuvenation	*E. cava*	Up-regulates HSP70, inhibits MAPK/NF-κB signaling and MMPs, reduces oxidative stress, promotes collagen deposition	Improves skin elasticity and photoaging	[167]
	Glucoraphanin-fortified kale	Delivers specific miRNAs to inhibit Smad7 and up-regulate collagen synthesis genes	Promotes collagen production and skin health	[178]
	*Phellinus linteus*	miR-CM1 inhibits Mical2, down-regulates MMP1 and oxidative stress markers, up-regulates COL1A2 and SOD	Prevents UV-induced photoaging	[49]
	Aloe vera	Activates the Nrf2/ARE signaling pathway, mitigating UV-induced oxidative stress and DNA damage	Protects against photoaging	[177]
	Ginseng	Inhibits the activation protein-1 signaling pathway, reduces ROS production	Prevents ultraviolet radiation and oxidative stress	[180]
	*Lentinus edodes*	Exhibits strong antioxidant and radiation protection properties	Prevents damage caused by infrared radiation	[181]
Anti-inflammatory and soothing repair	Turmeric	Reduces the expression of proinflammatory factors, enhances HO-1 activity	Alleviates skin inflammation, promotes repair	[33]
	*L. erythrorhizon* callus	Dose-dependently inhibits LPS-induced TNF-α production	Anti-inflammatory, soothes skin	[186]
	Goldenberry	Reduces M1 macrophage products and promotes polarization toward the M2 phenotype	Modulates inflammatory microenvironment	[193]
	Coriander	Internalized by skin tissue, promotes cell migration, scavenges ROS by up-regulating antioxidant enzymes, and alleviates inflammation	Accelerates wound healing	[185]
	Papaya	Contains multiple anti-inflammatory compounds, free radical scavenging, inhibition of various pathways	Exhibits broad-spectrum anti-inflammatory, antimicrobial, and antifungal potential	[187–192]
Skin whitening and spot reduction	*C. japonica*	Down-regulates the expression of MITF, TYR, TRP-1/2 genes and tyrosinase activity, inhibits MAPK/Akt/ERK pathways	Inhibits melanogenesis, brightens complexion	[203,204,206]
	*R. verniciflua* Stokes	Significantly reduces tyrosinase activity and melanin content	Skin whitening, lightens hyperpigmentation	[203,204]
	Yam bean	Promotes skin regeneration and modulates melanin metabolism to improve hyperpigmentation	Ameliorates hyperpigmentation and melasma	[208]
Skin barrier repair	Lavender	Scavenges ROS, targets Wnt/β-catenin and MAPK pathways via miR166 family, inhibiting inflammation and promoting collagen synthesis	Accelerates reepithelialization, repairs barrier	[179]
	Red onion	Scavenges ROS, enhances SOD activity, inhibits proinflammatory factors	Reduces oxidative stress and inflammation, promotes repair	[210]
Wound healing and regeneration	Coriander	Activates Nrf2 pathway to up-regulate antioxidant enzymes and promotes keratinocyte migration	Accelerates healing of full-thickness skin defects	[185]
	Lemon	Polarizes macrophages to the M2 phenotype, promotes angiogenesis and fibroblast migration	Promotes diabetic wound healing	[121]
	Wheatgrass	Activates integrin–FAK signaling to promote cell migration and up-regulates type I collagen expression	Promotes reepithelialization and matrix remodeling	[218]
	Dendrobium	Inhibits IL-1β expression in wound tissue, exerting anti-inflammatory effects to promote healing	Promotes wound healing by modulating inflammation	[214]
	Grapefruit	Enhances the tube-forming capacity of HUVECs by regulating VEGF-associated pathways	Promotes angiogenesis in wound beds	[215]
	Ginseng	Synergistically promotes the establishment of new vascular networks by modulating the angiopoietin-1/Tie2 signaling pathway	Provides nutritional and oxygen support for wound repair	[216,217]
	Edible kudzu	Regulates the TLR4/NF-κB pathway to induce macrophage polarization toward the M2 phenotype, synergistically inhibiting excessive inflammation	Accelerates repair by immunomodulation	[56,70]
Scar prevention and treatment	Rose	Improves scar texture and contraction, promotes the quality of tissue repair and aesthetic outcomes	Ameliorates traumatic and postacne scars	[164,224]
	Beetroot	Modulates the balance of collagen I/III and hyaluronan synthase 2 expression in fibroblasts and inhibits their migratory capacity	Suggests potential in regulating ECM homeostasis for scar modulation	[225]
Hair loss prevention and follicle regeneration	Shiitake mushroom	Inhibits NLRP3 inflammasome activation, reduces levels of IL-6 and IL-1β	Alleviates inflammation-mediated follicular damage	[231]
	Iris germanica rhizome	Activates Wnt/β-catenin signaling pathway, enhancing dermal papilla cell function.	Promotes hair follicle regeneration and cycle restart	[235]
	Papaya	Significantly inhibits nitric oxide synthesis and the expression of proinflammatory cytokines, up-regulate the anti-inflammatory cytokine	Modulates the perifollicular immune microenvironment	[187]
	Germinated hemp seed	Effectively antagonizes dihydrotestosterone-induced follicular damage, with superior protective effects compared to crude extract, indicating high bioavailability and therapeutic potential	Protects against androgen-mediated follicular injury	[234]

PLDEs: plant-derived exosome; HSP70, heat shock protein 70; MAPK, mitogen-activated protein kinase; NF-κB, nuclear factor κ light-chain enhancer of activated B cells; MMPs, matrix metalloproteinases; Nrf2, nuclear factor erythroid 2-related factor 2; ARE, antioxidant response element; TNF-α, tumor necrosis factor-α; IL, interleukin; HO-1, heme oxygenase-1; LPS, lipopolysaccharide; MITF, microphthalmia-associated transcription factor; TYR, tyrosinase; TRP, tyrosinase-related protein; ROS, reactive oxygen species; SOD, superoxide dismutase; FAK, focal adhesion kinase; NLRP3, NOD-, LRR- and pyrin domain-containing protein 3; FELNVs, fungal exosome-like nanovesicles; GelMA, gelatin methacryloyl; DAS, dialdehyde starch; VEGF, vascular endothelial growth factor; HUVECs, human umbilical vein endothelial cells; Ang-1, angiopoietin-1; TLR4, Toll-like receptor 4; CDENs, coriander-derived exosome-like nanoparticles; ECM, extracellular matrix; UV, ultraviolet; ERK, extracellular signal–regulated kinase; miRNAs, microRNAs.

## Challenges

Although PLDEs represent an emerging platform in cosmetics and regenerative medicine, their clinical translation faces critical bottlenecks, the most pressing of which is the lack of standardized preparation and characterization protocols. This absence leads to considerable batch-to-batch variations in purity, composition, and functionality, thereby limiting experimental reproducibility and hindering large-scale production. To move beyond this impasse, the field would benefit from adapting existing frameworks such as the minimal information for studies of EVs guidelines, which, although originally developed for mammalian EVs, provide valuable reference points for PLDEs—for example, by defining minimal characterization parameters (e.g., particle size, concentration, and purity indicators), recommending orthogonal techniques for validation, and emphasizing transparent reporting of experimental details to ensure reproducibility. Translating these principles to plant systems, while still evolving, offers a practical pathway toward harmonization. Beyond these technical hurdles, deeper questions remain unresolved, including the precise mechanisms of action, core active components, multicomponent synergistic effects, and in vivo metabolic kinetics of PLDEs. This knowledge gap restricts the ability to regulate therapeutic efficacy precisely and evaluate safety profiles reliably. Moreover, existing research remains largely confined to preclinical stages, with systematic human trial data lacking to validate actual efficacy and consistency. When coupled with the absence of long-term safety data and ongoing debates over regulatory classification under different frameworks, these challenges collectively pose substantial barriers to the transition of PLDEs from laboratory to clinical application. Addressing them will require a concerted effort to develop plant-specific characterization standards, harmonize regulatory definitions, and promote transparent reporting practices across the research community.

## Summary and Future Directions

As naturally occurring nanoscale vesicles and multifunctional bioactive carriers, PLDEs hold significant potential in cosmetic surgery. This review systematically summarizes the compositional characteristics, isolation methods, and engineering strategies of PLDEs, along with their mechanisms of action in skin antiaging, anti-inflammation, whitening, barrier repair, wound healing, and regeneration. PLDEs not only exhibit excellent biocompatibility, low immunogenicity, and high permeability but also enable precise regulation of the skin microenvironment by delivering plant-specific miRNAs, antioxidant metabolites, and signaling molecules via inhibiting the NF-κB/MAPK pathway, activating the Nrf2/ARE antioxidant pathway, and promoting collagen synthesis and angiogenesis.

Despite considerable promise, the clinical translation of PLDEs remains constrained by several interrelated challenges, including the absence of standardized protocols for isolation and characterization, limited understanding of their in vivo pharmacokinetics and mechanistic pathways, and a lack of robust clinical evidence confirming their safety and efficacy. Addressing these gaps will require a coordinated effort to establish harmonized standards for PLDE production and quality control, thereby enabling reproducibility and scalability. Further exploration using multiomics methods, in vivo tracking techniques, and artificial intelligence technology is warranted to elucidate the functional roles and synergistic interactions of key bioactive components in PLDEs. For instance, an artificial intelligence virtual EV digital model has been recently proposed. This model can intelligently predict the molecular composition (such as proteins, RNA, lipids, etc.), signal transmission mechanism, and targeting ability and drug loading efficiency of engineered and modified EVs [236]. Concurrently, engineering strategies, ranging from surface functionalization and drug loading optimization to membrane hybridization, offer avenues to enhance targeting capacity, stability, and therapeutic potency. To maximize translational relevance, future work should also focus on integrating PLDEs into advanced delivery platforms, including hydrogels, MNs, and 3D printed scaffolds to enable controlled release and sustained retention within cutaneous tissues. Ultimately, well-designed human trials should be conducted to evaluate the tolerability, efficacy, and clinical outcomes of PLDE-based interventions, providing the evidence base necessary to support their regulatory approval and broader adoption in aesthetic and regenerative medicine.

PLDEs represent the cutting-edge intersection of “green nanomedicine” and the field of aesthetic regeneration. Their further development is expected to drive innovative applications of naturally sourced nanoparticle formulations in cosmetic surgery and regenerative medicine.

## Data Availability

No datasets were generated or analyzed during the current study.
